# Functional Resilience against Climate-Driven Extinctions – Comparing the Functional Diversity of European and North American Tree Floras

**DOI:** 10.1371/journal.pone.0148607

**Published:** 2016-02-05

**Authors:** Mario Liebergesell, Björn Reu, Ulrike Stahl, Martin Freiberg, Erik Welk, Jens Kattge, J. Hans C. Cornelissen, Josep Peñuelas, Christian Wirth

**Affiliations:** 1 Department of Special Botany and Functional Biodiversity, University of Leipzig, Leipzig, Germany; 2 Escuela de Biología, Universidad Industrial de Santander, Cra. 27 Calle 9, 680002, Bucaramanga, Colombia; 3 Max-Planck-Institute for Biogeochemistry, Jena, Germany; 4 Department of Geobotany, University of Halle/Saale, Halle/Saale, Germany; 5 Systems Ecology, Dept. of Ecological Science, VU University, Amsterdam, The Netherlands; 6 CSIC, Global Ecology Unit CREAF-CSIC-UAB, 08193 Cerdanyola del Vallès, Barcelona, Catalonia, Spain; 7 CREAF, 08193 Cerdanyola del Vallès, Barcelona, Catalonia, Spain; 8 German Centre for Integrative Biodiversity Research (iDiv) Halle-Jena-Leipzig, Leipzig, Germany; Chinese Academy of Forestry, CHINA

## Abstract

Future global change scenarios predict a dramatic loss of biodiversity for many regions in the world, potentially reducing the resistance and resilience of ecosystem functions. Once before, during Plio-Pleistocene glaciations, harsher climatic conditions in Europe as compared to North America led to a more depauperate tree flora. Here we hypothesize that this climate driven species loss has also reduced functional diversity in Europe as compared to North America. We used variation in 26 traits for 154 North American and 66 European tree species and grid-based co-occurrences derived from distribution maps to compare functional diversity patterns of the two continents. First, we identified similar regions with respect to contemporary climate in the temperate zone of North America and Europe. Second, we compared the functional diversity of both continents and for the climatically similar sub-regions using the functional dispersion-index (FDis) and the functional richness index (FRic). Third, we accounted in these comparisons for grid-scale differences in species richness, and, fourth, investigated the associated trait spaces using dimensionality reduction. For gymnosperms we find similar functional diversity on both continents, whereas for angiosperms functional diversity is significantly greater in Europe than in North America. These results are consistent across different scales, for climatically similar regions and considering species richness patterns. We decomposed these differences in trait space occupation into differences in functional diversity vs. differences in functional identity. We show that climate-driven species loss on a continental scale might be decoupled from or at least not linearly related to changes in functional diversity. This might be important when analyzing the effects of climate-driven biodiversity change on ecosystem functioning.

## Introduction

Future global biodiversity scenarios and climate change projections predict dramatic species losses for many regions in the world [1,2]. With species loss, especially of plants as primary producers, important ecosystem functions may be altered, as these may depend on the characteristics of the extinct species and their interactions with the environment. In other words, species extinctions could reduce the functional diversity of entire floras and, as a consequence, lead to impoverished ecosystem functioning and a reduced ability to respond to future global changes if important pre-adapted species go missing [1,3].

Evidence for the effect of local species loss and subsequently reduced functional diversity on ecosystem functioning abounds for several ecosystems [4]. For example, experimental species loss and reduced functional richness have led to a reduction in numerous functions in grasslands [5] and forests [6]. Large-scale observational studies using forest inventory plots show reduced productivity, multifunctionality, and resilience after disturbance along species richness gradients from poly- to monocultures created by forest management [7]. In these examples, detection of functional diversity effects was possible because the richness gradients did not co-vary with environmental gradients owing either to the experimental or observational design.

Quite similarly, climate-driven extinctions during the recent geological history have created strong species richness gradients across continents within the same biome, i.e. in disparate regions with similar contemporary environment. A striking example of such a large-scale ‘geological experiment’ is the temperate tree flora of the Northern hemisphere, where–quoting Latham and Ricklefs [8]–“*within temperate latitudes*, *the mesic forests of eastern Asia have three times more tree species than forests in eastern North America and six times more than those in Europe*.” In our study, we focus on the comparison between Europe and North America. Despite a relatively high climatic similarity of both continents today, temperate forests host more tree species in North America than in Europe [8,9]. Since the tree flora on both continents has historically been very similar [10], this difference in species richness results almost certainly from more severe extinctions due to harsher glacial climates and postglacial dispersal limitation in Europe as compared to North America, especially during the Plio-Pleistocene glaciations 21,000 years ago [11].

Can such a massive reduction in species pool size by two thirds lower the functional diversity and thus potentially impair the functioning of forest ecosystems? In a random extinction scenario, as simulated in the vast majority of biodiversity-ecosystem functioning experiments, species go extinct irrespective of their functional characteristics. If species are evenly distributed in trait space, i.e. in the multidimensional space formed by functional trait axes [12], a random reduction from about 300 to 100 species would hardly change the extent of trait space occupation, but merely thin out the multidimensional ‘point cloud’ formed by the species pool. Consequently, we predict neither functional richness (the joint trait ranges) nor functional dispersion (the mean distance of species from the centroid of the ‘cloud’) to be strongly altered. However, real-world species extinctions are rarely random [13]. The glacial extinction of tree species in Europe affected species with particular ecological requirements. Cold-tolerant taxa such as *Betula sp*. are still widespread across Europe, while temperature-demanding taxa such as *Cupressus sp*. are restricted to the Mediterranean Basin and most moisture and warmth-demanding taxa have gone extinct (e.g. *Liquidambar sp*., *Caria sp*. [9]). As a likely consequence of this directed extinction process, European tree species are in general more drought-tolerant than the average American tree species [14] and the most frost-tolerant deciduous tree species in Eurasia, such as *Betula pubescens* and *Populus tremula*, grow under colder winter temperatures than their closest relatives in North America (e.g. *Betula papyrifera*, *Populus tremuloides*, *Populus balsamifera*).

Unlike under a random scenario, functional diversity may be lowered under a ‘non-random’ directed extinction scenario as species with a particular trait configuration are lost. Because environmental filters tend to take away species at the fringes of the species cloud in trait space [9,15,16], the overall extent of the trait space occupation, i.e. the functional richness of a flora [17,18] and with this the potential for functional diversity, may be reduced. While this has been demonstrated for a limited set of traits related to environmental tolerances of trees (i.e. climate envelope parameters [9]) and tested for continental phylogenetic diversity [10], the influence of the glacial extinction on the overall functional diversity of European and North American forests has not yet been investigated.

Here we ask, whether the climate-driven extinction of tree species during the last glaciations has led to a systematic reduction of functional diversity among European trees as compared to the functional diversity of North American trees. Specifically we hypothesize: (1) Because of the greater extinction of European species, the functional diversity–corrected for contemporary species richness and climate–should be smaller for the European than the North American tree flora (hereafter called: diversity change hypothesis). (2) Because of the directed extinction event, the trait space occupied by the European tree flora has been systematically shifted compared to the trait space occupied by North American trees, which has led to a change in the functional identity (i.e. identity change hypothesis).

To test these two hypotheses, we use a comprehensive trait matrix consisting of 26 traits that allow for a whole-plant perspective when investigating the effect of environmental filters on trees. Furthermore, many of these traits are so-called effect traits and are directly relevant for ecosystem functioning, such as life form, maximum height or leaf carbon to nitrogen ratio, which affect biogeochemical cycles [19]. Specifically, we compare the trait space occupied by 66 European tree species with that of its North American counterpart of 154 North American tree species. To do so, we first selected (currently) climatically similar regions in the temperate zone of North America and Europe. Second, we compared the functional diversity of both continents for climatically similar sub-regions using the functional dispersion-index (FDis [20]) and the functional richness index (FRic [18]). Third, we account in these comparisons for grid-scale differences in species richness, and, fourth, investigate the associated trait spaces using dimensionality reduction. Finally, we perform statistical tests for significance in changes in functional richness and identity and identify the responsible traits for these changes.

## Materials and Methods

### 2.1. Trait data

We assembled a trait matrix for 26 traits including 154 North American (126 angiosperms, 28 gymnosperms) and 66 European tree species (56 angiosperms, 10 gymnosperms) from the temperate zone. A single species, *Juniperus communis*, occurred on both continents. We defined a tree as a woody plant growing taller than 6 m anywhere within its geographical range. We excluded lianas from the analysis. We selected the most important species based on abundance and dominance, as derived for Europe from Bohn et al. [21] and Food and Agriculture Organization of the United Nations [22] and for North America from the US Forest Service’s Forest Inventory and Analysis (FIA) program [23]. Owing to gaps in the availability of trait and species distribution information, as well as to methodological restrictions of the studied regions on both continents (see below), the full list of ecologically important species was reduced to a final number of approximately 50% in North America and 30% in Europe (S1 Table).

Trait values for North American species were provided by Stahl et al. [24], whereas trait values for European species were extracted from various databases and the literature (S2 Table). In accordance with Stahl et al. [24] we aimed for a comprehensive characterization of the tree species’ functional potential and thus based the selection of traits on the following criteria: (1) They reflect adaptations to cope with stress (water, light, nutrient limitation), disturbance (wind, snow, herbivory) and competition [19]. (2) They are causally related to one of the three fitness components growth, reproduction or survival [25]. (3) They represent relevant functions of different plant organs: leaf, stem, below-ground and regenerative traits allowing for a whole-plant perspective. Note that we did not include climate envelope traits as in Svenning [9].

We harmonized trait data to standard SI-units and aggregated them to the species level resulting in species-specific mean trait values. This is based on ample evidence that interspecific variation tends to be much greater than intraspecific variation for most traits [26]. We aggregated continuous traits by their median due to its robustness against outliers. Ordinal traits were aggregated by their mean since the number of replicates for this data type was usually low. In case of few data points with high variability the results for median values would be conservative and may obscure variability in the data. Nominal traits were aggregated by their mode. The final trait matrix consisted of 7 continuous traits, 4 binary traits, 9 ordinal traits, 4 nominal traits and 2 multi-choice nominal traits and included 14% missing values (Table 1 For trait descriptions see S3 Table). The missing values were reduced to 6% by complementing continuous and ordinal traits with the mean values of the respective genera. Finally, we used the Gower distance measure, which has been shown to be robust for this low amount of missing data [27], to construct a dissimilarity matrix based on which functional diversity indices and principle coordinate analysis were calculated.

**Table 1:** Description of trait classes, units and trait level abbreviations.

	trait name		data type	Trait levels or units	abbreviation
*leaf traits*					
	leaf area		continuous	mm^2^	l.area
	leaf arrangement		nominal	Alternate, Whorled, Opposite, Spirally, Shortshoot, alternate and/or opposite, other	l.alternate, l.whorled, l.opposite, l.spirally, l.short.shoot, l.alternate/opposite, l.other
	leaf carbon:nitrogen ratio		continuous	mg*g^-1^	leaf.cn
	leaf composition		binary	Simple, composite	l.simple, l.composite
	leaf margin		ordinal	0 = entire, 1 = toothed and/or entire, 2 = toothed, 3 = lobed	leaf.mar,
	leaf type		nominal	evergreen needle, evergreen scale, evergreen broadleaved, deciduous broadleaved, deciduous needle, evergreen and/or deciduous broadleaved	l.evgr.needle, l.evgr.scale, l.evgr.broad, l.decid.broad, l.decid.needle, l.evgr/ decid.broad
	specific leaf area		continuous	cm^2^*g^-1^	sla
*plant level traits*					
	fire resistance (flammability)		binary	0 = not fire resistant, 1 = fire resistant	fire.res
	growth form		nominal	Tree, Shrub, Tree or shrub	Tree, Shrub, tree/shrub
	growth rate		ordinal	1 = slow, 2 = moderate, 3 = rapid	growth.rate
	lifespan		continuous	a	life.span
	maximum height		continuous	m	height
	nitrogen fixation		binary	not N-fixing, N-fixing	n.fix
	potential allelopathy		binary	0 = not allelopathic, 1 = allelopathic	allelo
	resprout ability after disturbance		binary	0 = no resprout ability after disturbance, 1 = resprout ability after disturbance	resp.dist
	resprout ability after fire		ordinal	0 = none, 1 = low, 2 = medium, 3 = high	resp.fire
	toxicity		ordinal	0 = none, 1 = low, 2 = medium, 3 = high	tox
*root and wood traits*					
	are tracheids present?		binary	0 = no tracheids, 1 = tracheids present	tracheids
	bark surface		ordinal	1 = smooth, 2 = between smooth and medium, 3 = medium, 4 = between medium and rugged, 5 = rugged	bark.surf
	conduit type and arrangement (porosity)		multichoice nominal	ring, semi.ring, diffuse	porosity
	rooting habit		nominal	tap root, shallow root, variable root habit	tap.root, shallow.root, var.root
	wood density		continuous	kg*m^-3^	wood.dens
*reproduction traits*					
	dispersal syndrome		multichoice nominal	animal, wind, gravity, water	animal.disp, wind.disp, gravity.disp, water.disp
	seed mass		continuous	mg	seed.mass
	seed spread rate		ordinal	1 = slow, 2 = moderate, 3 = rapid	seed.spread
	vegetative spread rate		ordinal	1 = slow, 2 = moderate, 3 = rapid	veg.spread

### 2.2. Spatial data

**Fig 1:**
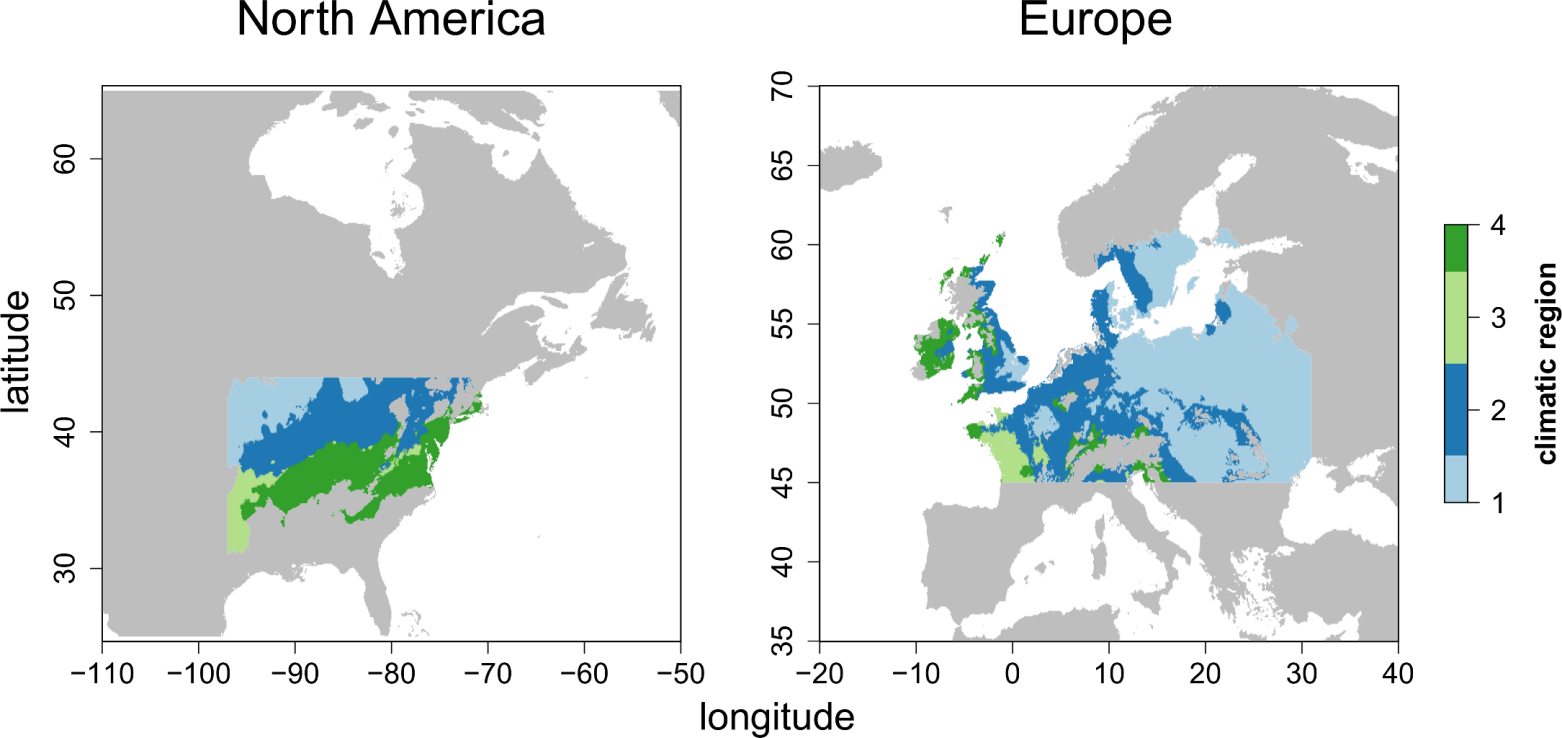
Map of the four bioclimatic regions, chosen as the result of the PCA of 19 bioclimatic variables intersected with the temperate climate zone as defined by Walter & Breckle [30].

We assembled distribution maps for all tree species in the trait matrix. Range maps for North American trees were obtained from U.S. Geological Survey [28]. Range maps for European species were assembled from different sources (S1 File). We transferred the range maps to a raster with a 5 arc minute resolution (approx. 10 by 10 km) from which we derived a species co-occurrence matrix considering all land area potentially suitable for the species considered. To minimize the confounding effect of potential differences in topographic heterogeneity between the continents, we excluded all grid cells with an elevation greater than 1000 m from subsequent analyses. Since species distributions and co-occurrences not only depend on altitudinal elevation but may also depend on the spatial variation in topographic height, we compared the topographic variability on both continents statistically (S2 File). To analyze only climatically similar regions of North America and Europe, we conducted a Principle Component Analysis (PCA) using 19 bioclimatic variables obtained from worldclim.org [29]. The first two PCA axes explained 80% of the total variance and were used to subdivide the climate space of both continents into four climatically similar regions, which in turn were restricted to the temperate region as defined by Walter & Breckle [30] over Europe and North America (Fig 1). We then compared functional diversity patterns within each of these regions separately to achieve comparability.

By restricting the four study regions to the temperate zone, potentially important tree species were excluded from further analyses. Thus, trait and distribution information was assembled for non-temperate tree species from the list of ecologically important species. Information on these trees was gathered in exactly the same way as described for tree species in the four regions. We ran all analyzes using this enhanced set of species, which can be viewed as a test of robustness against a potential sampling bias between the two continents (S3 File).

Finally, to test the robustness of the results against differences in climate classification systems we also ran the analysis replacing the Walter & Breckle [30] with the Koeppen-Geiger climate classification [31,32] (S4 File).

### 2.3. Statistical analysis

To account for the strong phylogenetic differences in morphological and physiological traits between gymnosperms and angiosperms [24] we conducted all analyses separately for each of the two groups. The analyses for gymnosperms were based on 22 traits because the traits wood porosity, leaf compoundness, presence of tracheids and nitrogen fixation did not vary within this group, while 26 traits were used for the analysis of angiosperms. In the following, we present the different analysis steps that we used to address the outlined hypotheses:

1. **Species dissimilarities in trait space.** To compare the functional diversity of angiosperms and gymnosperms, respectively, between the sub-regions of both continents we analyzed the occupation patterns of species in multi-dimensional trait space. To calculate the distribution of species in trait space, we used the Gower distance, which retains Euclidian properties and was calculated following Pavoine et al [33]. Where necessary, continuous traits were square-root- or log-transformed beforehand to obtain normal distribution. We standardized all traits to zero mean and unit variance.
2. **Calculation of functional diversity indices.** Dissimilarity matrices derived from the Gower distance were used to calculate functional diversity indices at the continental scale, at the sub-regional scale (i.e. considering the four climatically similar regions described in Section 2.2) and at the grid-cell scale with a 5 arc min resolution. We calculated the following functional diversity indices: (1) Functional dispersion (FDis [20]) measures the dispersion of species in trait space as the mean distance to the centroid. (2) Functional richness (FRic [18]) measures the volume of the n-dimensional trait space (S5 File). FRic and FDis are complementary: While FRic measures the extent to which the trait space is filled, FDis measures how this space is filled while giving a more conservative measure of its size.
3. **Testing for differences in functional dispersion.** To test for differences in the functional dispersion of species in trait space between continents and sub-regions we used a test of multivariate homogeneity of group dispersions (betadisper function in the R package vegan [34];[35]), which was carried out on the Gower dissimilarity matrix. First, the average distance of all group members to the group centroid in multivariate space was calculated. In our case, the group was the continental origin of the species. Subsequently, the model residuals were permutated to generate a permutation distribution of F-values to test if one of the two groups was more variable. We accounted for small sample sizes with a $sqrt/\frac{n}{n-1}$ correction [36].
4. **Testing for differences in functional identity.** In order to test for differences in the location of the centroid of the trait space between continents and sub-regions, we used a permutational Multivariate Analysis of Variance (perMANOVA) on the Gower distance matrix (vegan package in R [34]). Significance tests were done using F-tests based on sequential sums of squares from permutations of the raw data [34]. We used permutations of the raw data since they show better small sample characteristics compared to permutations on residuals.
5. **Trait-based interpretation.** For the visualization of the results and their interpretation in terms of the underlying traits we used biplots from PCoA analysis. We note that these plots are for interpretation purpose only and are based on the reduced trait space spanned by the first two PCoA axes and do not consider the full information content as in the analysis steps 2–5. For visualizing the differences in species trait space occupation between the two continents, we used a 2-dimensional kernel density estimation (ks package in R [37]) highlighting the density centers of the trait spaces.

## Results

### Gymnosperms

**Fig 2:**
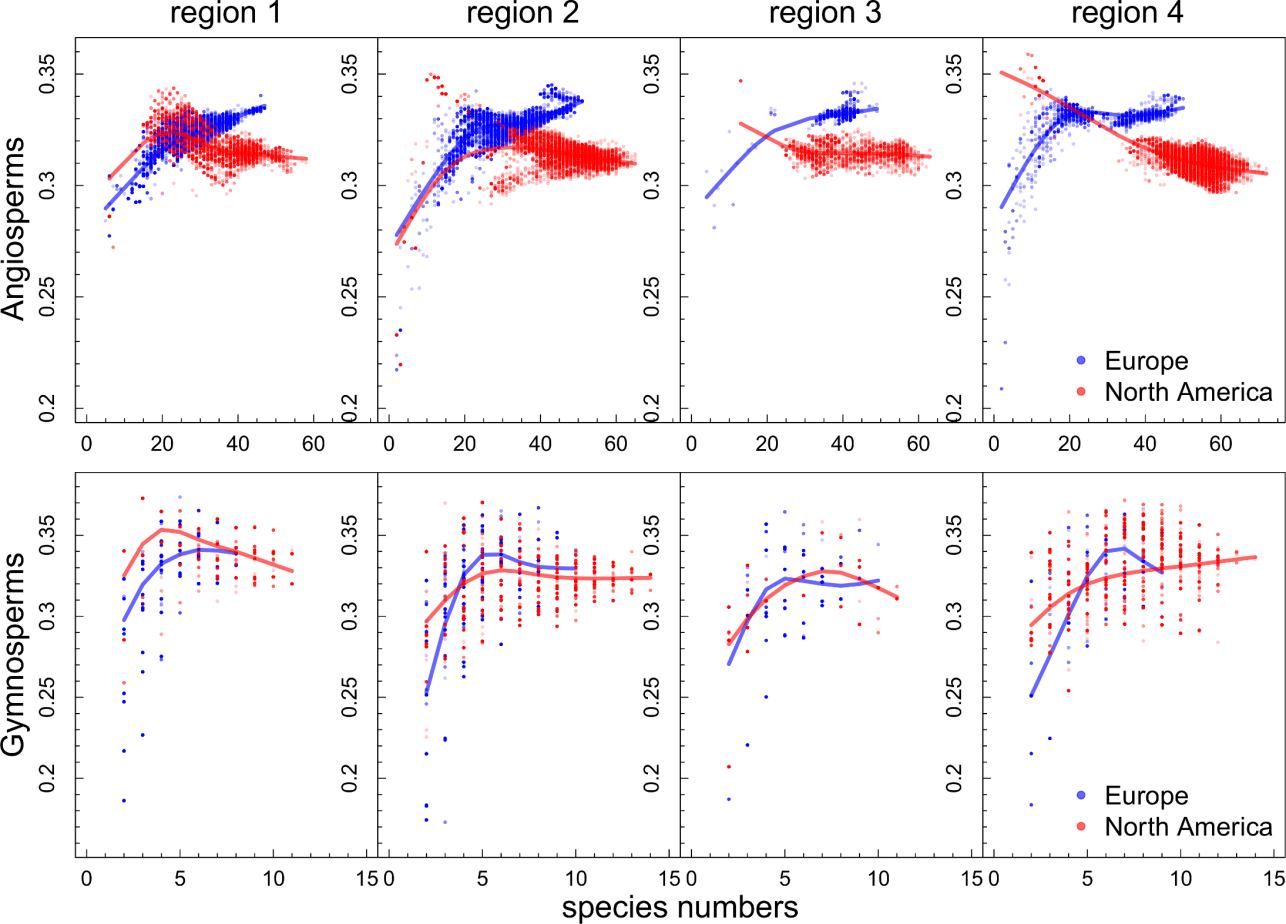
The relationship of species richness and functional dispersion for European and North American gymnosperm and angiosperm communities, based on 5arcmin species distribution maps.

**Fig 3:**
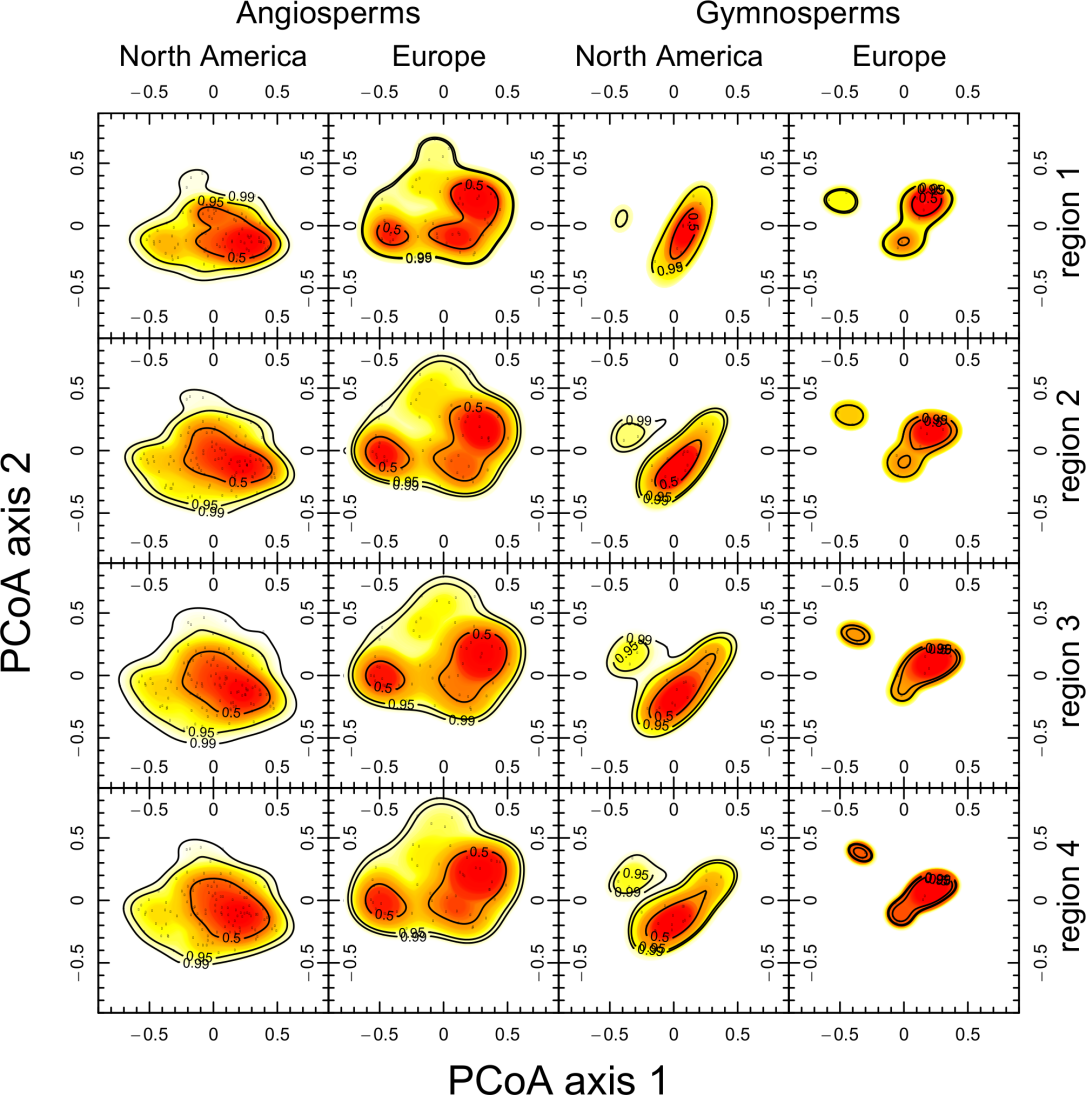
Kernel density estimations of the first two PCoA-axes based on a Gower distance matrix of 26 traits for 126 North American and 56 European angiosperms, as well as for 29 North American and 11 European gymnosperms in four climatic regions separately.

**Fig 4:**
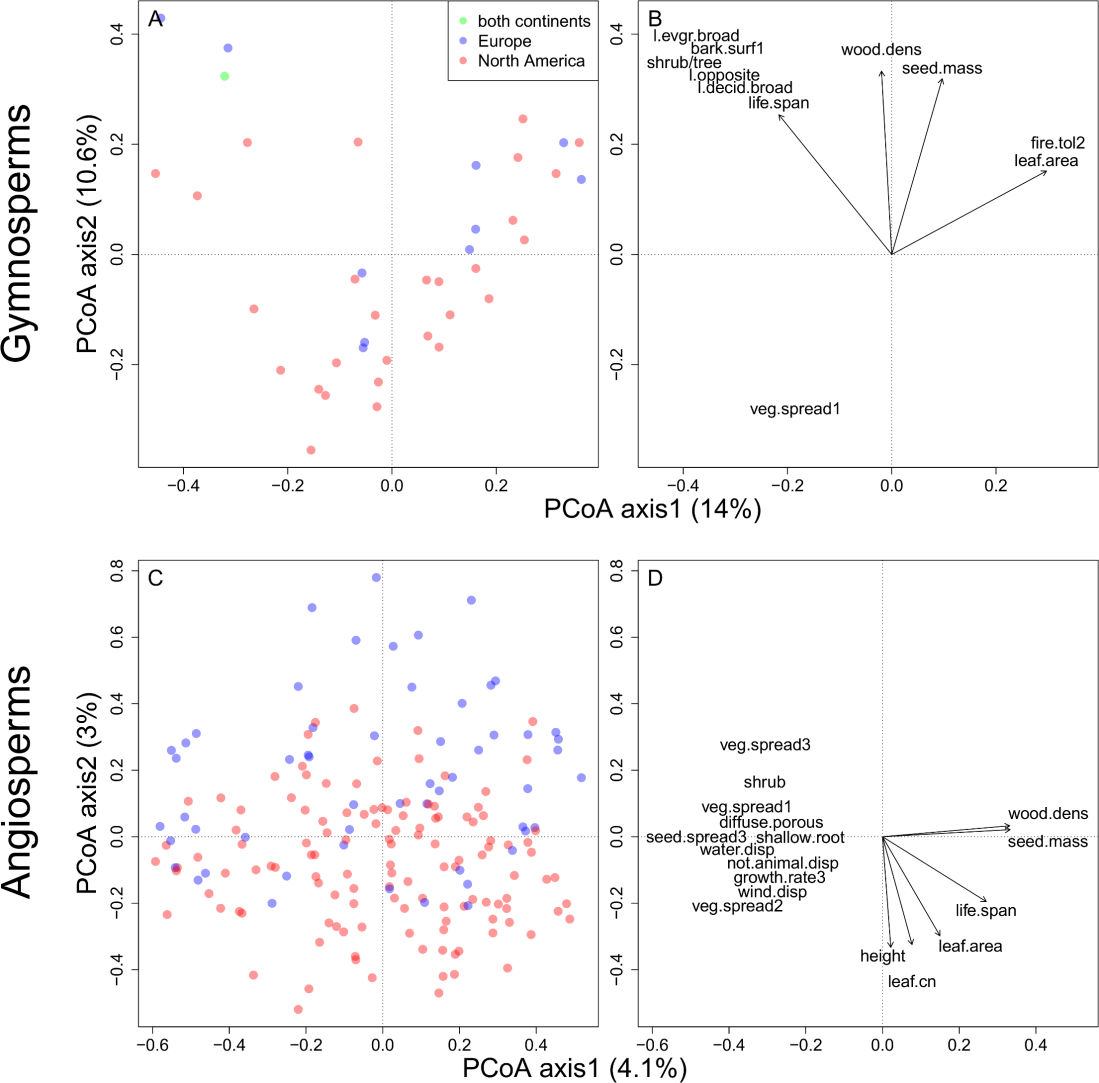
PCoA ordination plot showing (A) distances among 29 North American and 11 European woody gymnosperm species based on 22 traits for the first two axes. (C) PCoA ordination plot showing distances among 126 North American and 56 European woody angiosperm species based on 26 traits for the first two axes. In (B and D) significant correlations (p, 0.001) with a loading of min +/- 0.25 of traits on the first two PCoA axes are represented as arrows (see Table 1 for abbreviations) for gymnosperms and angiosperms respectively; the lengths of the arrows are proportional to their correlation coefficient, and they point in the direction of most rapid change; nominal traits were dummy coded before correlation.

For the gymnosperms, the functional dispersion of species in trait space is not different between Europe and North America. This pattern is consistent for the continental, the sub-regional as well as the grid-cell scale (Fig 2 and Fig 3). This means that neither in the location of the centroid nor in the dispersion of species in trait space a significant difference between the continents can be detected (Table 2). At the scale of grid-cells a similar pattern emerges revealing broad overlap of FDis among grid-cells between both continents when considering FDis and species richness simultaneously (Fig 2). In other words, for plant assemblages of similar species richness the functional dispersion for gymnosperms on both continents is similar. The same results were obtained from the analyses without climatic restrictions (S3 File) and using the Koeppen-Geiger classification for temperate regions (S4 File). Considering functional richness (FRic) as a measure of the size of the trait space results in a similar pattern (S5 File). However, when visually inspecting the trait space occupation at the sub-regional scale with respect to the first two PCoA axes, one may argue that North American species disperse more than European species (Fig 3). The PCoA—biplot reveals that this difference may arise from the second axis and is caused by a large number of North American *Pinaceae* species with small leaf blades, short tree lifespan, seed mass and wood density which are able to reproduce vegetatively (Fig 4, for biplot with species names see S6 File). European gymnosperms do not cover this region of the trait space. Instead *Juniperus oxycedrus and Taxus baccata* occupy the trait space at the opposite side of the second axis, which relates to long tree lifespan, high wood density and high seed mass. Thus, the second axis reflects the life strategies of resource conservative stress tolerators and acquisitive resource exploiters.

**Table 2:** Summary of the permutation test for differences in multivariate homogeneity of group dispersions (Functional dispersion) between the continents based on 999 permutations, and the perMANOVA for differences in variance between the functional clouds based.

			*multivariate homogeneity of group dispersions (FDis)*		*variance between the functional clouds (perMANOVA)*	
class	region	df	*F*	*p*.*val*	*pseudo-F*	*p*.*val*
gymnosperms	All regions pooled	1	1.0517	0.319	1.5081	0.116
	Region1	1	0.8951	0.316	1.272	0.226
	Region2	1	0.8174	0.372	1.492	0.121
	Region3	1	0.9752	0.33	1.4035	0.157
	Region4	1	0.8093	0.357	1.4783	0.126
angiosperms	**All regions pooled**	**1**	**17.35**	**0.001**	**7.9521**	**<0.001**
	**Region1**	**1**	**12.941**	**0.001**	**5.9826**	**<0.001**
	**Region2**	**1**	**14.481**	**0.001**	**7.1096**	**<0.001**
	**Region3**	**1**	**15.63**	**0.002**	**7.5206**	**<0.001**
	**Region4**	**1**	**15.929**	**0.001**	**7.5793**	**<0.001**

### Angiosperms

In contrast, for the angiosperms we find significant differences in trait space occupation between North America and Europe at the continental, the sub-regional and the grid-cell scale (Fig 2 and Fig 3). At the grid-cell scale we find a rapid increase of FDis with species richness in all North American subregions (Fig 2) peaking at intermediate species richness levels and subsequently declining. While at low species richness levels this pattern is ambiguous, European tree assemblages at higher species richness levels consistently exhibit a greater FDis. This result is consistent with the findings at the continental and sub-regional scale, where the dispersion of European tree species is significantly greater than the dispersion of North American species (Table 2). The result is also consistent for FRic (S5 File), for the Koeppen-Geiger classification of temperate regions (S4 File) and for the whole continents without climatic restrictions (S3 File). Because of two density centers in the European trait space as contrasted to a single density center within the North American angiosperms (Fig 3), the locations of the trait space centroids also differ significantly between the continents (Table 2).

These two density centers within the trait space of European angiosperms arise from the functional differences of specific taxa: *Castanea sp*., *Juglans sp*. and *Quercus sp*. in Europe exhibit a long lifespan, high wood density, low growth rate and high seed mass (Fig 4, for biplot with species names see S6 File). These species show low seed spread rate and do not reproduce vegetatively. The North American pendants are *Carya sp*., *Gymnocladus sp*., as well as *Quercus sp*. On the opposite side of the first axis lie short-lived wind dispersed species with high growth rate, high vegetative and seed spread rate and low seed mass. On both continents these are species of the genera *Populus*, *Betula* and *Salix*. Thus, the first axis reflects the difference between resource acquisitive pioneers and resource conservative species, whereas the second axis separates the species pools of both continents. It opposes relatively small, short lived trees with small leaves (e.g. *Euonymus europaeus*, *Prunus mahaleb*) from Europe with tall, long lived trees with big leaves and high C:N-ratio from North America (e.g. *Liriodendron sp*., *Ulmus sp*., *Fraxinus sp*.). Thus, the second axis reflects the life strategies of over- and understorey vegetation.

## Discussion

In this study, we compared the trait space occupation patterns of gymnosperms and angiosperms between Europe and North America taking a whole-plant perspective considering a comprehensive set of functional traits. We utilized traits that define interspecific variation associated with climatic conditions (i.e. response traits such as leaf morphology or seed mass) as well as traits affecting ecosystem functions (i.e. effect traits such as wood density or growth rate). Despite more severe climate-driven extinctions in Europe during glacial periods, gymnosperms exhibit similar functional diversity on both continents, whereas angiosperms in Europe–en contra our expectation–exhibit a greater functional diversity as compared to North America. These results are consistent across different scales, on the whole continental scale, for climatically similar regions and considering species richness patterns. Below, we decompose these differences in trait space occupation into differences in functional diversity vs. differences in functional identity.

### Continental differences in functional diversity

Based on our finding we reject our first hypothesis that a larger species pool as a consequence of less severe climate-driven extinctions should have resulted in a higher functional diversity in North America. For gymnosperms, there is neither a visible difference in FDis between the continents at the grid-cell scale nor a statistical one at the sub-regional or continental scale. In contrast, angiosperms in Europe even exhibit a higher FDis across all spatial scales than North American angiosperm trees. This is interesting, because we consider nearly three times as many gymnosperm species (28:10) for North America compared to Europe and more than twice as many angiosperm species (128:56). Our findings clearly contradict the common assumption that species richness and functional diversity should be highly correlated and that a declining species pool translates into declining functional diversity as a consequence of selection effects and accidental loss of functionally distinct species [4]. Our findings suggest that the loss of species does not simply translate into a loss of functional diversity and that this can vary according to the organismic group under investigation.

### Continental differences in functional identity

Our second hypothesis stated that because of a directed climate-driven extinction event the trait space of European trees should have been systematically reduced within a particular region resulting in a shift of the trait space centroid and hence in the functional identity of the whole flora. Especially we expected the extinction of moisture and warmth demanding taxa such as *Liquidambar sp*., *Carya sp*. would have left a portion of the trait space unoccupied leading to a shift towards more cold adapted taxa. For example, the long-lived pioneer *Liriodendron tulipifera* [38] died out after the last glaciations in Europe as a consequence of its climatic requirements. Physiologically *L*. *tulipifera* resembles typical pioneer species with high light demand and fast growth rate. But unlike other pioneers it grows tall and lives comparably long [39,40]. Long-lived pioneers recover carbon at fast rates and keep it for a very long time thus increase the landscape level carbon storage [39]. We expect the extinction of *L*. *tulipifera* would have left a portion of the trait space unoccupied.

Such a shift in the trait space was observed for angiosperms, but not for the gymnosperms.

While the gymosperms of both continents overlap almost entirely along the first PCoA axis, there was a tendency towards a systematic shift along the second axis (Fig 4), even though this was not significant. Here, North American members of the Pinaceae family (*Picea glauca*, *Picea mariana*, *Abies fraseri*, *Larix laricina*, *Tsuga sp*.) are opposed to the European species *Taxus baccata* and *Juniperus oxycedrus*. With the exception of *Tsuga sp*. these genera are widespread on both continents and do not point to a climatically driven extinction event. Nevertheless, the European gymnosperms are shifted upwards along the second PCoA axis suggesting a tendency for higher drought tolerance as indicated by tree or shrub-like habit, high wood density and large seed mass (cf. Svenning [9]; Stahl et al. [24]). North American gymnosperms, in contrast, are characterized by taller stature, short tree lifespan, lower seed mass and wood density and vegetative reproduction. The tendency for larger seed mass, as we could show for European gymnosperms, is often associated with an increased resistance to hazards and pests as well as to the ability for fast germination and growth [41]. It is also an adaptation to tolerate shading [42]. Similarly, high wood density is positively correlated with pathogen-resistance and mechanical stability [43]. Moreover, denser wood increases the resistance to drought-induced xylem embolism [44] and is thus associated to drought-resistance [45]. Consequently, the second PCoA-axis reflects two different life strategies: fast growing exploitative pioneers with low wood density and seed mass are opposed to slow growing stress tolerators with higher wood density and seed mass. On this strategy axis, North American species are shifted towards the resource exploiter-side while European gymnosperms appear to be more resistant to unfavorable environmental conditions. This result adds additional support to the hypotheses of Svenning [9], Manthey and Box [14] and Eiserhardt *et al*. [10] that European tree floras are more tolerant of drought and cold stress, as a result of climatic filtering.

For the angiosperms, we find a statistically significant shift of the trait space along the second PCoA axis. Since this axis however only explains very little variance we are cautionary in terms of the interpretation. The second axis opposes lower stature trees with low leaf C:N-ratio from Europe (e.g. *Ilex aquifolium*, *Laurus nobilis*, *Prunus mahaleb*) with tall, long-living trees with big leaf blades and high leaf C:N-ratio in North America (*Liriodendron sp*., *Ulmus sp*., *Fraxinus sp*., *Quercus sp*.*)*. The axis reflects the life strategies of tall, light demanding overstorey vegetation vs. smaller, rather shade tolerant trees, which tend to belong to the understorey. On this strategy axis North American angiosperms are shifted towards the overstorey side while European species are shifted towards the resistant, understorey side of this axis. Overstorey species develop greater values for maximum height, which is a proxy for growth and development after disturbance [41] or for a high longevity. Moreover, taller plants intercept more radiation and could thus potentially realize high photosynthetic assimilation/respiration rates [46] and develop higher growth rates [47].

*Ulmus sp*., *Fraxinus sp*, *Quercus sp*. as well as *Prunus sp*. and *Ilex sp*. are common genera on both continents. As a single exception *Liriodendron sp*., once widespread in Europe, died out after the last glaciations. It is the only genus that can be related to a climatically driven extinction event. While Stahl et al. [24] found no clear correlation of the above mentioned traits with drought or frost tolerance among North American species, European angiosperms—as already seen for the gymnosperms- tend to be more resistant to unfavorable environmental conditions. As for the gymnosperms, these results are in line with the hypotheses of Svenning [9], Manthey and Box [14] and Eiserhardt *et al*. [10].

### Limitations

Our findings are subject to limitations: First, while North American tree species distributions were consistently documented and harmonized by U.S. Geological Survey [28], distribution maps for European species come from multiple source of differing quality. Some regions in Europe have been thoroughly sampled, whereas others might be under-sampled or even missing. A bias in the species sample is inevitable in such a continental analysis. Nevertheless, because we expect the under-sampling to occur predominantly in Europe, we would mainly expect an increase in functional diversity in Europe as compared to North America, further strengthening our findings.

Second, finding strictly equivalent climatic regions between Europe and North America is a challenge. Although several bioclimatic classification systems postulate equal climatic zones on both continents [30,32,48], the contemporary climate of the temperate zone in Europe cannot exactly match that in North America, where many regions are consistently warmer, wetter and of different seasonality. Therefore, we used a straightforward and simple approach based on principle component analysis of 19 bioclimatic variables. Because our results are consistent across different scales and diversity levels, as well as against different climate classifications of temperate climates, we consider them robust.

Third, although we restricted our sample regions by altitude, environmental heterogeneity may have an influence on functional differences between the continents. S2 File shows that the analyzed European regions have a higher topographic heterogeneity as compared to the North American regions. Nevertheless, our results are robust across different scales and extends of analyzed regions.

Fourth, the functional differences between both continents may decline over time, as remaining species in a restricted community can get the functions of the extinct species by accelerated adaptation or evolution on the basis of phenotypic plasticity, epigenetics, gene flow and genotypic plasticity [49]. Considerable genetic variation and gene flow may be present in many natural populations. For many species however, gene flow between populations may be critically low because of the effects of habitat fragmentation.

### Implications for conservation and the study of biodiversity-ecosystem processes

Since the earliest empirical contributions to the field of biodiversity-ecosystem functioning research numerous studies have shown that biodiversity matters for ecosystem functioning. There is broad agreement that biodiversity, be it expressed as species richness or functional diversity, is generally positively correlated with the magnitude and stability of many ecosystem functions, e.g. litter decomposition [50] and productivity, in plant communities [5,7]. Multifunctionality, a high level of operation across several ecosystem functions, is particularly sensitive to biodiversity [51,52]. Some studies state, that species richness and functional diversity are surrogates [4] and the proposed mechanisms relating species richness to ecosystem functioning such as complementarity of resource use have a strong functional basis [53], as they require plants to be different with respect to particular functional traits (e.g. rooting depth, phenological niches, etc.).

However, the existing evidence refers to the local scale and the richness gradients in experimental or observational settings typically vary between 1 and 16, rarely up to 60 species, whereas differences between regional floras are in numbers of hundreds of species. Moreover, the species comprising regional floras do not coexist locally, but form communities rarely exceeding a richness of 30 species per hectare. It is thus not valid to translate findings from experiments or forest inventories based on small plots to the regional grid-scale.

However, there is no consistent explanation on how species with their trait configuration contribute to ecosystem functioning. Bunker et al. [54] showed that changes in the mean community value of a single trait can modify whole ecosystem processes. Using different scenarios of selective harvesting in a tropical forest ecosystem, they revealed a decrease or increase of carbon storage up to 75% depending on the mere modification of community wood density. Hence, even changes in a single trait may lead to dramatic changes in ecosystem processes.

Evidence that changes in species richness are likely to change functional diversity have led to the call for biodiversity protection, especially under human induced global change [55]. As we have shown, the climate-driven loss in species richness might be decoupled or at least not linearly related to changes in functional diversity of entire regions. This makes predictions about changes in ecosystem functioning caused by climate change inherently difficult.

But we must not understand this as an argument against biodiversity preservation. Indeed, the insurance hypothesis [4] postulates that species rich communities most likely host functionally similar species which can buffer against changes in species composition under changing environmental conditions. As a consequence, it predicts that both resistance to and resilience against disturbances, is higher in species rich environments. In other words, species loss does not necessarily lead to impoverished ecosystem processes when the underlying species pool can buffer against it. This hypothesis is empirically underpinned by the study of Balvanera et al. [56], who found that the effects of biodiversity on ecosystem processes play out on different organizational levels: changing biodiversity in their study has had a strong influence on the community level, but the effect on the ecosystem level was neither clearly positive nor negative and depended on the specific type of process under investigation. Our findings demonstrate that the stronger climatically driven loss of tree species in Europe than in North America during the last glaciations has not led to a lower functional diversity in Europe. Instead, a high local and regional species richness seems to buffer against functional impoverishment via functionally redundant species.

## Supporting Information

S1 File Species distribution data: processing and references. (DOCX)

S2 File Environmental heterogeneity analyses. (DOCX)

S3 File Analyses without restriction to the temperate zone. (DOCX)

S4 File Analyses for the temperate zone according to the Koeppen-Geiger classification. (DOCX)

S5 File Functional Richness analyses. (DOCX)

S6 File Additional information on the PCoA—analyses. (DOCX)

S7 File Species co-occurrences. (ZIP)

S8 File Permission to use data from third parties under CC BY 4.0. (DOCX)

S1 Table Species sample. (DOCX)

S2 Table Trait data references. (XLSX)

S3 Table Trait description. (DOCX)

S4 Table Trait data from open sources. (XLSX)
