# Supplementary material for: Functional Resilience against Climate-Driven Extinctions – Comparing the Functional Diversity of European and North American Tree Floras
Source: PLoS One. 2016 Feb 5;11(2):e0148607. doi: 10.1371/journal.pone.0148607 (PMC4743854; doi:10.1371/journal.pone.0148607)
Supplement: S1 File — (DOCX) [file pone.0148607.s001.docx]

# Appendix S1 File - Species distribution data: processing and references

*Species distribution data processing by Martin Freiberg*

The number of species in the species pool of each plot was estimated as the potential species richness from tree species distribution maps. The distribution maps were provided by Martin Freiberg from the Botanical Gardens, Leipzig and Erik Welk from the Botanical Gardens, Halle, and compiled for the FunDivEUROPE project (<http://www.fundiveurope.eu>) by Mario Liebergesell to estimate the potential species richness at 5 arc minute resolution. Nomenclature follows the Atlas Florae Europaea.

*Species distribution data processing by Erik Welk*

The tree distribution data were compiled from floristic literature supplied with occurrence data from web based data sources to obtain total geographic range data sets. In published atlases and floras complete or partial range distribution maps are given. These maps were scanned, georeferenced and digitized in ArcMap 9.1 (see List of plant distribution data sources). The compilation of the distribution maps from partial and regional data sources included also verbal descriptions of distribution limits and lists of occurrence localities. These place names were searched with the help of the software GEOLocate 3.22 (Rios & Bart, 2010). Combining all available data the species ranges were generalized to represent areas of similar likelihood of the species occurring in a species specific mean density. Single isolated occurrence locations were omitted in this generalization in order to obtain contiguous geographical areas. List of plant distribution data sources for woody species used by E.W. to compile the geographic distributions, web based data sources were accessed in the past, mainly in 2012.

reference: Rios, N. E. & Bart, H. L. (2010). GEOLocate (Version 3.22), [Computer software]. Belle Chasse, LA: Tulane University Museum of Natural History.

Figure 1: Species richness map of the whole species sample (gymnosperms + angiosperms).

Table 1: References of species distribution data.

| **author** | **region** | **reference** |
| --- | --- | --- |
| Martin Freiberg | Austria - Kärnten | Hartl H et al (1992) Verbreitungsatlas der Farn- und Blütenpflanzen Kärntens, 227 pages |
| Martin Freiberg | Austria - Salzburg | Wagner H and Heislmeyer P (editor) (1987) Sauteria, Beiträge zu Geobotanik, Pflanzensystematik und Floristik, Abakus Verlag Salzburg, 996 maps |
| Martin Freiberg | Austria - Steiermark | Maurer W (1996-2006) Flora der Steiermark, Vol 1ff, 311 pages |
| Martin Freiberg | Austria - Tirol | Polatschek A and Neuner W (1997-2013) Flora von Nordtirol, Osttirol & Vorarlberg, Vol 1-7, Tiroler Landesmuseen-Betriebsgesellschaft, Innsbruck |
| Martin Freiberg | Belgium | Observatoire de la Faune, de la Flore et des Habitats (OFFH), La biodiversité en Wallonie, available at: http://biodiversite.wallonie.be/fr/atlas-permanent.html?IDC=807 |
| Martin Freiberg | Bulgaria | Assyov B and Petrova A (editors) (2006) Conspectus of the vascular Flora of Bulgaria, distribution maps and floristic elements, 3rd edition, 452 pages |
| Erik Welk | Czech Republic | Slavík B. [ed.] (1997): Květena České republiky. 5. Praha: Academia. |
| Erik Welk | Czech Republic | Slavík B., Smejkal M., Dvořáková M., Grulich V. [eds] (1995) Květena České republiky. 4. Academia, Praha, 529 p. |
| Erik Welk | Czech Republic | Štěpánková J., Chrtek J. jun. & Kaplan Z. [eds.] (2010): Květena České republiky. Sv. 8. Praha: Academia. |
| Martin Freiberg | Estonia | Kukk T and Kull T (editos) (2005) Atlas of the Estonian Flora, Institute of Agricultural and environmental Sciences of the Estonian University of Life sciences, Tartu, 471 pages |
| Erik Welk | Finland | Kasviatlas (2012). Atlas of the Vascular Flora of Finland. Available at: http://www.luomus.fi/kasviatlas |
| Martin Freiberg | France | Olivier L et al (editor) (1995) Livre rouge de la Flore menacée de France, Tome 1: Espèces prioritaires, Institut d’Ecologie et de Gestion de la Biodiversite Service du Patrimoine naturel. 486 pages |
| Martin Freiberg | France - Alpes Maritimes | Franco C, Flore des Alpes Maritimes, available at: http://flore06.voila.net/ |
| Martin Freiberg | France - Alsace | Société Botanique d'Alsace, Atlas de la Flore d’Alsace, available at: http://www.atlasflorealsace.com/ |
| Martin Freiberg | France - Auvergne | Antonetti P et al (2006) Atlas de la Flore d’Auvergne, Conservatoire botanique national du Massif central, 984 pages |
| Martin Freiberg | France - Bassin Parisien | Conservatoire Botanique National du Bassin Parisien, , available at: http://cbnbp.mnhn.fr/cbnbp/ |
| Martin Freiberg | France - Cantal | Sapaly J (1982) Atlas de la Flore du cantal, Paris, Ministere de l’Environmnet, 188 planche |
| Martin Freiberg | France - Drome | Garraud L (2003) Flore de la Drome: Atlas e Floristique, 926 pages |
| Martin Freiberg | France - Franche-Comté | angiofc (2005-2010), Fleurs  de  Franche-Comté et alentours, available at: http://angiofc.free.fr/esplat.php |
| Martin Freiberg | France - Franche-Comté | Conservatoire Botanique National de Franche-Comté, La flore franc-comtoise : atlas en ligne - Cartes de répartition des espèces présentes en Franche-Comté, available at: http://www.conservatoire-botanique-fc.org/cartes-taxons/form-espece.php |
| Martin Freiberg | France - Haute-Alpes | Société Alpine de Protection de la Nature - Groupe Flore, Atlas en ligne de la flore du départemnet des Hautes-Alpes, Version 1.30, available at: http://www.bdflore05.org/ |
| Martin Freiberg | France - Haute-Normandie | Centre régional de phytosociologie, l'inventaire de la flore sauvage de la Haute-Normandie, available at: http://digitale.cbnbl.org/digitale-rft/site/Atlas/Atlas_HN/hn.jsp |
| Martin Freiberg | France - Isère | Gentiana, Société botanique dauphinoise D.Villars (2007-2008), La flore d’Isère, available at: http://www.gentiana.org/site:flore |
| Martin Freiberg | France - Lorraine | Association des Botaniste Lorraines, Floraine, available at: http://www.floraine.net/ |
| Martin Freiberg | France - Provence | Association TELA BOTANICA Institut de Botanique 163, Rue Auguste Broussonnet 34090 Montpellie, Tela Botanica - the french botany network, available at: http://www.tela-botanica.org/page:chorologie_carte?format=html&module=chorologie |
| Martin Freiberg | France - Pyrenes | Flore des Pyrénées et de France, available at: http://www.afleurdepau.com/Flore.htm |
| Martin Freiberg | France - Rennes | Dairad L (2005) Atlas de la Flore d’Ille-et-Vilaine, Editions Siloe, 612 pages |
| Martin Freiberg | France - Var | Cruon R (editor) (2008) Le Var et sa Flore, naturalia publications, 451 pages |
| Martin Freiberg | France - Vaucluse | Vaucluse's Orchids, available at: http://pm.blais.pagesperso-orange.fr/list.orch.vau.html |
| Erik Welk | generic | Anonymous, ed. 1969. Flora of Transbaikalia. Tomsk: Tomsk University. 149 p. (In Russian) |
| Erik Welk | generic | Artportalen (2012) Reporting System for Vascular Plants and Fungi. Available at: http://www.artportalen.se/default.asp |
| Erik Welk | generic | Artsdatabanken (2012) Nasjonal kunnskapskilde for biologisk mangfold. Available at: http://www.artsdatabanken.no/ |
| Martin Freiberg | generic | Botanical Society of the British Isles, BSBI Vascular Plant Records, available at: http://www.bsbimaps.org.uk/atlas/atlas_abspecies_select.php |
| Erik Welk | generic | Geydeman, T.S. (1986) Opredelitel’ vysshikh rasteniy Moldavskoy SSR. Stiinta, Kishinev. |
| Erik Welk | generic | Hultén, E. (1971) Atlas of the distribution of vascular plants in northwestern Europe. Generalstabens Litografiska Anstalts Förlag, Stockholm. |
| Erik Welk | generic | INPN (2012) Inventaire National du Patrimoine Naturel. Available at: http://inpn.mnhn.fr/accueil/index?lg=en |
| Erik Welk | generic | Jalas, J. & Suominen, J. eds. 1972-1994. *Atlas Florae Europaeae: Distribution of Vascular Plants in Europe*. The Committee for Mapping the Flora of Europe and Societas Biologica Fennica Vanamo, Helsinki, Finland. |
| Erik Welk | generic | Maevskii, P.F. (1954) Flora of middle belt of the European part of the USSR. Selkhosgiz, Moscow. |
| Erik Welk | generic | Meusel H, Jäger, E.J., Weinert, (1965) Vergleichende Chorologie der zentraleuropäischen Flora. Text u. Karten. Bd. 1. Fischer, Jena. |
| Erik Welk | generic | Meusel, H., Jäger, E.J. (1992): Vergleichende Chorologie der zentraleuropäischen Flora. Text u. Karten. Bd. 3. - Gustav Fischer Verlag Stuttgart New York. |
| Erik Welk | generic | Meusel, H., Jäger, E.J., Rauschert, S., Weinert, E. (1978): Vergleichende Chorologie der zentraleuropäischen Flora. Text u. Karten. Bd. 2. - Fischer Jena. |
| Martin Freiberg | generic | Royal Botanic Garden Edinburgh (1998), Flora Europaea, available at: http://rbg-web2.rbge.org.uk/FE/fe.html |
| Erik Welk | generic | SILENE (2012) Système d'Information et de Localisation des Espèces Natives et Envahissantes. Available at: http://silene.eu/index.php?cont=accueil |
| Erik Welk | generic | SOPHY (2012) Banque de Données Botaniques et Ecologiques. Available at: http://sophy.u-3mrs.fr/sophy.htm |
| Erik Welk | Germany | Benkert, D., Fukarek, F. & Korsch, H. [ed.] (1996) Verbreitungsatlas der Farn- und Blütenpflanzen Ostdeutschlandp. Fischer, Jena. 615 p. |
| Martin Freiberg | Germany | Bundesamt für Naturschutz (BfN), FloraWeb-Daten und Informationen zu Wildpflanzen und zur Vegetation Deutschlands, available at: http://www.floraweb.de/index.html |
| Erik Welk | Germany | Haeupler, H. & Schönfelder, P. [ed.] (1988) Atlas der Farn- und Blütenpflanzen der Bundesrepublik Deutschland. Ulmer, Stuttgart. 768 p. |
| Erik Welk | Germany | Haeupler, H., Jagel, A., Schumacher, W. (2003): Verbreitungsatlas der Farn- und Blütenpflanzen in Nordrhein- Westfalen. 616 p. |
| Erik Welk | Germany | Lang, W. & Wolff, P. (1993) Flora der Pfalz. Verbreitungsatlas der Farn- und Blütenpflanzen für die Pfalz und ihre Randgebiete. Veröff. Pfälz. Ges. Förd. Wiss. Speyer 85: 444 p. |
| Erik Welk | Germany | Oberdorfer, E. (1983): Pflanzensoziologische Exkursionsflora. - Eugen Ulmer Verlag Stuttgart. 5 Aufl. |
| Erik Welk | Germany | Raabe, E.W. (1987) Atlas der Flora Schleswig-Holsteins und Hamburgs. Neumünster, 654 p. |
| Erik Welk | Germany | Sauer, E. (1993) Die Gefäßpflanzen des Saarlandes. Aus Natur und Landschaft, Sonderband 5, Saarbrücken, 708 p. |
| Erik Welk | Germany | Schönfelder, P., Bresinsky, A. [ed.] (1990) Verbreitungsatlas der Farn- und Blütenpflanzen Bayerns. Ulmer, Stuttgart, 752 p. |
| Erik Welk | Germany | Schumacher, W. [ed.] (1995) Atlas der Farn- und Blütenpflanzen des Rheinlandes. Bonn, 353 p. |
| Erik Welk | Great Britain | Perring, F.H., Walters, S.M. (eds.) (1993): Atlas of the British Flora. 3rd ed. repr. - Melksham (Redwood Press). |
| Martin Freiberg | Iberian Peninsula | Carlos A (Director), Anthos. Spanish plants information system, available at: http://www.anthos.es/ |
| Martin Freiberg | Iberian Peninsula - Aragon | unknown authors, available at: http://webipe.ipe.csic.es/floragon/mapas/ |
| Martin Freiberg | Iberian Peninsula - Asturia | Government of the Principality of Asturias, Asturias environmental net, available at: http://www.asturias.es/portal/site/medioambiente |
| Martin Freiberg | Iberian Peninsula - Burgos | Sáenz A, García-López and Sanz M (editors) (2006) Atlas de la Flora Vascular Silvestre de Burgos, Junta de castilla y León, Caja rural de Burgos, 924 pages |
| Martin Freiberg | Iberian Peninsula - Catalonia | de Càceres M, Quadrada R, Moreno J and Martí D, Dept.Biologia Vegetal (U.B.), Biodiversity bank of Catalonia, available at: http://biodiver.bio.ub.es/biocat/index.jsp |
| Martin Freiberg | Iberian Peninsula - Extremadura | Palacios Gonzáles MJ et al (2010) Catálogo regional de especies vegetales amenazadas de Extremadura, Collecion Medio Ambiente, Junta de Extremadura, 448 pages |
| Martin Freiberg | Iberian Peninsula - Pais Vasco | Aizpuru I et al (2002) Estudio de la flora vascular amenazada de los arenales de la Comunidad Autónoma del País Vasco, 111 pages |
| Martin Freiberg | Iberian Peninsula - Portugal | Flora digital de Portugal , available at: http://jb.utad.pt/especie |
| Martin Freiberg | Iberian Peninsula - Sorio | unknown authors (2000), Catálogo florístico de la provincia de Soria, available at: http://www.jolube.net/mapas/mapas.htm |
| Martin Freiberg | Iberian Peninsula - Valencia | Generalitat Valenciana, Conselleria de Infraestructuras, Territorio y Medio Ambient, Banko de Datos Biodiversitat Comunidad Valenciana, available at: http://bdb.cth.gva.es |
| Erik Welk | Italy | Flora Italiana (2012). Flora Italiana. Available at: http://www.homolaicus.com/scienza/erbario/utility/floraitalica/flora/index.htm |
| Erik Welk | Italy | Poldini, L. (1991) Atlante corologico delle piante vasculari nel Friuli-Venezia Giulia. Direzione Regionale delle Foreste e dei Parchi, Udine. |
| Erik Welk | Kazakhstan | Pavlov, N.V., ed. 1956-1966. Flora of Kazakhstan. V. 1-9. Alma-Ata: AN Kaz. SSR. 754 p. (In Russian) |
| Erik Welk | Kroatia | FCD (2012) Flora Croatica Database. Available at: http://hirc.botanic.hr/fcd/ |
| Martin Freiberg | Latvija | SIA "Gandrs", Visu internetenciklopēdijas "Latvijas daba" tekstu un ilustratīvo materiālu autortiesību īpašnieks ir Askolds Kļaviņš, available at: http://www.latvijasdaba.lv/augi/sistematiskais-raditajs/ |
| Erik Welk | Netherlands | Mejden, R.. van der, Plate, C.L. & Weeda, E.J. (1989): Atlas van de Nederlandse Flora. 3. Minder zeltzame en algemene sorten. Leiden: Onderzoekinstituut Rijksherbarium / Hortus botanicus; Voorburg / Heerlen: Central Bureau voor de Statistiek. |
| Erik Welk | Netherlands | Mennema, J. u.a. (1985): Atlas van de Nederlandse Flora. Band 2: Zeldzame en vrij zeldzame planten. - Utrecht: Bohn, Scheltema & Holkema. |
| Erik Welk | Netherlands | Mennema, J., Quené-Boterenbrood, A.J. & Plate, C.L. (Hrsg.) (1980): Atlas of the Netherlands Flora. 1. Extinct and very rare species. - The Hague-Boston-London: Dr. W. Junk BV Publ. |
| Erik Welk | North America | Critchfield, W. B., & Little, E. L. (1966). *Geographic distribution of the pines of the world* (Vol. 991). US Department of Agriculture, Forest Service. |
| Erik Welk | North America | Little Jr, E. L. (1971). Atlas of United States trees. Volume 1. Conifers and important hardwoods. Miscellaneous publication 1146. *US Department of Agriculture, Forest Service, Washington, DC*. |
| Erik Welk | North America | Little, E. L. (1977). Atlas of United States Trees. Volume 4. Minor Eastern Hardwoods. Miscellaneous Publication 1342. *US Department of Agriculture, Forest Service, Washington, DC, US*. |
| Erik Welk | North America | Little, E. L., & Viereck, L. A. (1971). *Atlas of United States trees* (Vol. 5). US Dept. of Agriculture, Forest Service. |
| Erik Welk | North America | Prasad, A. M., & Iverson, L. R. (1999). *A climate change atlas for 80 forest tree species of the eastern United States*. Northeastern Research Station, USDA Forest Service. |
| Martin Freiberg | North America | USDA, NRCS (2011) The PLANTS Database. National Plant Data Team, Greensboro, NC 27401-4901 USA. Retrieved from http://plants.usda.gov |
| Martin Freiberg | Poland | Snowarski M (2002-2014), Atlas of vascular plants of Poland, available at: http://www.atlas-roslin.pl/index.html |
| Erik Welk | Russia | Blagoveshchenskii A.V., ed. 1966. Keys to plants of Moscow Region. Moscow: Nauka. 368 p. (In Russian) |
| Erik Welk | Russia | Fedorov A.A., ed. 1981. Keys to higher plants of Crimea. Leningrad: Nauka. 380 p. (In Russian) |
| Erik Welk | Russia | Galushko, A.I. 1978. Flora of the Northern Caucasus. V. 1. Rostov-na-Donu: Rostov University. 318 p. (In Russian) |
| Erik Welk | Russia | Galuško, A.I. (1980) Flora Severnogo Kavkaza. Isd. Rostowskogo Univ., Rostow. |
| Erik Welk | Russia | Grigor'evskaya, A.Ya. 2000. Flora of Voronezh town. Voronezh: Voronezh University. 198 p. (In Russian) |
| Erik Welk | Russia | Grossgejm, A.A. (1949) Opredelitel‘ rastenij Kavkaza. Sovj. Nauk, Moskow. |
| Erik Welk | Russia | Gubanov I.A., Kiseleva K.V., Novikov V.S., Tikhomirov V.N. 2002. The illustrated keys to plants of Middle Russia. V. 1. Moscow: KMK. 526 p. (In Russian) |
| Erik Welk | Russia | Kharkhevich S.S., ed. 1985. Vascular plants of the Soviet Far East. Leningrad: Nauka. V. 1. 446 p. (In Russian) |
| Erik Welk | Russia | Klirosova V.P., Noskova T.S., eds. 1975. Keys to plants of Kirov Region. Part 2. Kirov: Kirov Pedagogical institute. 304 p. (In Russian) |
| Erik Welk | Russia | Koroleva A.S., Krasnoborov I.M., Pen'kovskaya, E.F. 1973. Keys to plants of the Novosibirsk Region. Novosibirsk: Nauka. 368 p. (In Russian) |
| Erik Welk | Russia | Malyshev, L.I. & Peshkova, G.A. eds. 1979. Flora of Central Siberia. V. 1. Novosibirsk: Nauka. 536 p. (In Russian) |
| Erik Welk | Russia | Minyaev N.A., ed. 1970. Synopsis of flora of Pskov Region. Leningrad: Leningrad University. 176 p. (In Russian) |
| Erik Welk | Russia | Orlova N.I. 1997. Key to higher plants of the Vologda Region. Vologda: Rus'. 262 p. (In Russian) |
| Erik Welk | Russia | Popov, M.G. 1957. Flora of Middle Siberia. V. 1. Moscow-Leningrad: AS SSSR. 556 p. (In Russian) |
| Erik Welk | Russia | Ramenskaya M.L. 1960. Keys to vascular plants of Karelia. Petrozavodsk: Karelian Publishing House. 486 p. (In Russian) |
| Erik Welk | Russia | Shishkin, B.K., ed. 1955. Flora of the Leningrad Region. V. 1. Leningrad: Leningrad University. 288 p. (In Russian) |
| Erik Welk | Russia | Tikhomirov V.N., ed. 1975. Synopsis of flora of Konspekt Meshchera, Ryazan'. Moscow: Lesnaya promyshlennost'. 326 p. (In Russian) |
| Erik Welk | Russia | Tolmachev A.I., ed. 1966. Keys to plants of Primorskii Territory and Amur Region. Moscow-Leningrad: Nauka. 492 p. (In Russian) |
| Erik Welk | Russia | Tolmachev, A.I., ed. 1964. Arctic Flora of the USSR. V. 2. Graminea. Moscow-Leningrad: Nauka. 272 p. (In Russian) |
| Erik Welk | Russia | Tsvelev, N.N. (2000) Keys to the vascular plants of North-West Russia (Leningrad, Pskov and Novgorod Regions). SPbSCPA press, Saint-Petersburg. |
| Erik Welk | Russia | Tzvelev N.N., ed. 2001. Flora of East Europe. St.Petersburg. 670 p. |
| Erik Welk | Russia | Voroshilov, V.N. 1966. Flora of the Soviet Far East (the synopsis with the tables for species identification). Moscow: Nauka. 478 p. (In Russian) |
| Martin Freiberg | Scandinavia | Anderberg A and Anderberg A-L, Den virtuella floran, available at: http://linnaeus.nrm.se/flora/welcome.html |
| Martin Freiberg | Slovakia | Goliasova K and Michalkova E (editors) (2006) Flora Slovenska, Vol 4.2 – 5.3, Bratislava |
| Martin Freiberg | Slovenia | Nejc J et al. (2001) Gradivo za Atlas flore Slovenije. Miklavz na Dravskem polju. 158 pages |
| Erik Welk | Spain | Anthos (2012) Information System of Plants in Spain. Available at: http://www.anthos.es |
| Erik Welk | Spain - catalania | Bolós, O., & Vigo, J. (1995) Flora dels Paisos Catalans, Vol. 3. Editorial Barcino, Barcelona |
| Martin Freiberg | Switzerland | Wohlgemuth T, Boschi K and Longatti P (1999-2001), swiss web flora, Version 2.02, available at: http://www.wsl.ch/land/products/webflora/floramodul1-de.html |
| Erik Welk | Tatarstan | Bakin O.V., Rogova T.V., Sitnikova A.P. 2000. Vascular plants of Tatarstan. Kazan: Kazan University. 496 p. (In Russian) |
| Erik Welk | Turkmenistan | Nikitin V.V., Geldikhanov A.M. 1988. Keys to plants of Turkmenistan. Leningrad: Nauka. 670 p. (In Russian) |
| Erik Welk | Udmurtia | Baranova O.G., Il'minskikh N.G., Puzyrev A.N., Tuganaev V.V. 1992. The synopsis of flora of Udmurtia. Izhevsk: Udmurtian Univ. 140 p. (In Russian) |
