# Supplementary material for: Functional Resilience against Climate-Driven Extinctions – Comparing the Functional Diversity of European and North American Tree Floras
Source: PLoS One. 2016 Feb 5;11(2):e0148607. doi: 10.1371/journal.pone.0148607 (PMC4743854; doi:10.1371/journal.pone.0148607)
Supplement: S2 File — (DOCX) [file pone.0148607.s002.docx]

# Appendix S2 File: Environmental/topographic heterogeneity of both continents

1. MATERIALS AND METHODS

We calculated summary statistics with the topographic information on the temperate regions in Europe and North America. We chose two approaches to quantify environmental heterogeneity: 1) Since the analyzed regions on both continents cover different extents of landmass, we sampled both continents to the same number of grid cells 999 times and used Wilcoxon signed-rank tests to test for differences between the sampled mean values and standard deviations in altitude between the continents. We used bootstrap tests with 999 permutations on the W-statistics to infer the central tendencies and the robustness from these samples. 2) Altitudinal maps have a resolution of 5 arc min which equals an area of app. 30 x 30 km close to the equator. We aggregated these high resolution maps to the resolution of 1 degree which is app. 100 x 100 km close to the equator (3 x 3 grid cells of 5 arc min resolution form one grid cell of 1 degree resolution) and used the standard deviation of the aggregated grid cells as a measure of spatial heterogeneity. We sampled the standard deviation-values of both continents to the same number and compared the mean values with Wilcoxon signed-rank tests. We used bootstrap tests with 999 permutations on the W-statistics to infer the central tendencies and the robustness of these samples.

2. RESULTS

*summary statistics*

The sample of the North American temperate region is on average 20 m (mean) to 80 m (median) higher in altitude than the European sample (Table 1). The distribution of altitudes differs between both continents (Figure 1).

Table 1 Summary statistics of topographic information on the analyzed regions.

|  | Europe | North America |
| --- | --- | --- |
| Min [m] | -6 | -22 |
| 1st Quantile [m] | 70 | 121 |
| Mean [m] | 198 | 223.5 |
| Median [m] | 142 | 218 |
| 3rd Quantile [m] | 254 | 311 |


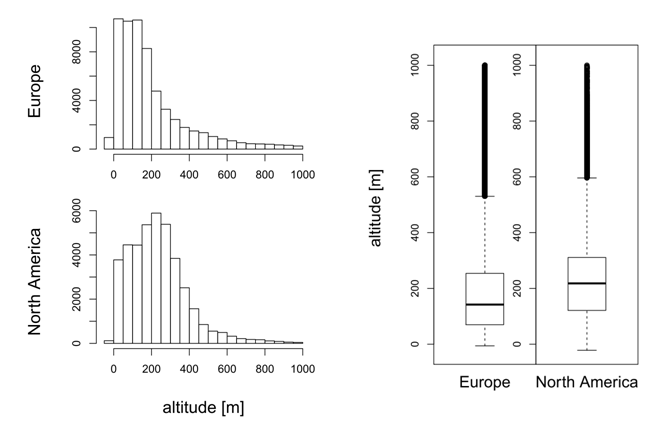


Figure 1 Histograms and Box-Whisker-Plots for the analyzed regions.

*First test for environmental heterogeneity*

The mean altitude of grid cells (5 arc min resolution) is significantly higher in Europe than in North America in subregion 1 and 2 (Table 1). In subregion 3 and 4, as well as for the whole temperate region, the mean altitude is higher in North America than in Europe. However, the standard deviation around these mean values is significantly higher in Europe than in North America for all subregions and for the whole temperate region (Table 2).

Table 1 First test of environmental heterogeneity for the mean values of altitude in 5 arc min grid cells.

|  |  | | **permutation test of 999 Wilcoxon-signed rank tests of differences between the altitude of both continents** | | | **Confidence interval of the normal distribution for the mean W-statistic** | |  |
| --- | --- | --- | --- | --- | --- | --- | --- | --- |
|  | **mean altitude Europe [m]** | **mean altitude North America [m]** | **mean W-statistic** | **bias** | **mean St.-Error** | **2.50%** | **97.50%** | **significance** |
| **area1** | 308.9 | 162.8 | 4025777 | 14.85973 | 995.2463 | 3828019 | 4223177 | **p < 0.001** |
| **area2** | 261.8 | 248.4 | 46759225 | -42.00207 | 2592.142 | 46255393 | 47266726 | **p < 0.001** |
| **area3** | 112.9 | 231.2 | 3151836 | 0.2489545 | 55.35026 | 3141226 | 3162680 | **p < 0.001** |
| **area4** | 220.1 | 220.3 | 8259805 | 10.32182 | 477.4031 | 8165781 | 8354428 | **p < 0.001** |
| **all regions** | 198 | 223.5 | 631400000 | 1053.302 | 45738.72 | 630809085 | 636310074 | **p < 0.001** |

Table 2 First test of environmental heterogeneity for the standard deviation of altitudes in 5 arc min grid cells.

|  |  |  | **permutation test of 999 Wilcoxon-signed rank tests of differences between the altitude of both continents** | | | **Confidence interval of the normal distribution for the mean W-statistic** | |  |
| --- | --- | --- | --- | --- | --- | --- | --- | --- |
|  | **sd Europe [m]** | **sd North America [m]** | **mean W-statistic** | **bias** | **mean St.-Error** | **2.50%** | **97.50%** | **significance** |
| **area1** | 124.2447 | 71.37785 | 4026422 | 81.51919 | 3173.818 | 3834588 | 4218197 | **p < 0.001** |
| **area2** | 228.821 | 107.8128 | 46763323 | 93.06622 | 7869.331 | 46277710 | 47242226 | **p < 0.001** |
| **area3** | 119.6064 | 100.8107 | 3152146 | -1.220509 | 169.9644 | 3141969 | 3162889 | **p < 0.001** |
| **area4** | 182.2205 | 205.6607 | 8261476 | 56.68444 | 1526.031 | 8166969 | 8359512 | **p < 0.001** |
| **all regions** | 191.7573 | 154.8825 | 633466939 | -262.9277 | 46831.29 | 630645123 | 636413468 | **p < 0.001** |

*Second test for environmental heterogeneity*

In Europe grid cells of an app. area of 120 x 120 km have an intrinsic altitudinal difference of 66 m. In North America this difference/ heterogeneity is lower with a mean value of 45 m (Table 3).

Table 3 Second test of environmental heterogeneity for the intrinsic standard deviation of altitudes in grid cells of 1 degree resolution.

|  |  |  | **permutation test of 999 Wilcoxon-signed rank tests of differences between the altitude of both continents** | | | **Confidence interval of the normal distribution for the mean W-statistic** | |  |
| --- | --- | --- | --- | --- | --- | --- | --- | --- |
|  | **Mean sd Europe [m]** | **Mean sd North America [m]** | **mean W-statistic** | **bias** | **mean St.-Error** | **2.50%** | **97.50%** | **significance** |
| **Temperate region** | 66.1 | 45.0 | 51900.84 | 2.125034 | 35.17492 | 49659.85 | 54085.2 | **p < 0.001** |

4. DISCUSSION

Our results suggest a stronger environmental heterogeneity, as measured by altitude, in Europe than in North America.
