# Supplementary material for: Functional Resilience against Climate-Driven Extinctions – Comparing the Functional Diversity of European and North American Tree Floras
Source: PLoS One. 2016 Feb 5;11(2):e0148607. doi: 10.1371/journal.pone.0148607 (PMC4743854; doi:10.1371/journal.pone.0148607)
Supplement: S3 File — (DOCX) [file pone.0148607.s003.docx]

# Appendix S3 File - Analysis undertaken on whole continental scale

1. MATERIALS AND METHODS

1.1. Trait data

Since we restricted our main analyses to the temperate region of both continents, some non-temperate species from our original species-dataset were omitted from further analyses. Hence we rerun the whole analyses without restricting the study regions to the temperate zone. We could therefore enhance our species set by 50 % (100 more tree species) as compared to the original analyses. These additional species were sampled in exactly the same way as described in the methods-section of the manuscript and fulfill the same requirements as those species in the main experiment (larger than 6 m in height, being in our list of economically important species, etc.).

In fact, we assembled a trait matrix for 26 traits including 246 North American (164 angiosperms, 82 gymnosperms) and 74 European tree species (63 angiosperms, 11 gymnosperms) from the whole continent. A single species, *Juniperus communis*, occurred on both continents. The species were chosen according to the same criteria as in the main experiment.

In addition to the species set from the main experiment the following 54 North American gymnosperms were included in the analyses: *Abies amabilis, Abies bracteata, Abies grandis, Abies lasiocarpa, Abies magnifica, Abies procera, Chamaecyparis lawsoniana, Chamaecyparis nootkatensis, Cupressus arizonica, Cupressus bakeri, Cupressus macrocarpa, Cupressus sargentii, Juniperus californica, Juniperus deppeana, Juniperus monosperma, Juniperus occidentalis, Juniperus osteosperma, Juniperus pinchotii, Juniperus scopulorum, Larix lyallii, Larix occidentalis, Picea breweriana, Picea engelmannii, Picea sitchensis, Pinus albicaulis, Pinus aristata, Pinus attenuata, Pinus balfouriana, Pinus cembroides, Pinus clausa, Pinus contorta, Pinus coulteri, Pinus engelmannii, Pinus flexilis, Pinus jeffreyi, Pinus lambertiana, Pinus longaeva, Pinus monophylla, Pinus monticola, Pinus muricata, Pinus ponderosa, Pinus quadrifolia, Pinus radiata, Pinus sabiniana, Pinus torreyana, Pseudotsuga macrocarpa, Pseudotsuga menziesii, Sequoia sempervirens, Taxus brevifolia, Thuja plicata, Torreya californica, Torreya taxifolia, Tsuga heterophylla, Tsuga mertensiana.*

*Larix kaempferi was included in the European dataset.*

*The following 38 North American angiosperms were included in the analyses: Acer glabrum, Acer grandidentatum, Acer macrophyllum, Aesculus californica, Alnus rhombifolia, Alnus rubra, Arbutus menziesii, Betula occidentalis, Cercocarpus ledifolius, Cornus nuttallii, Fraxinus latifolia, Fraxinus velutina, Juglans californica, Juglans hindsii, Lithocarpus densiflorus, Olneya tesota, Platanus racemosa, Populus angustifolia, Populus fremontii, Populus trichocarpa, Prosopis pubescens, Prunus emarginata, Quercus agrifolia, Quercus arizonica, Quercus chrysolepis, Quercus douglasii, Quercus emoryi, Quercus engelmannii, Quercus gambelii, Quercus garryana, Quercus grisea, Quercus hypoleucoides, Quercus kelloggii, Quercus lobata, Quercus oblongifolia, Rhizophora mangle, Robinia neomexicana, Umbellularia californica.*

*The following 7 European angiosperms were included in the analyses: Cydonia oblonga, Platanus orientalis, Punica granatum, Quercus faginea, Quercus macranthera, Quercus pontica, Syringa vulgaris,*

1.2. Spatial data

All species distribution maps arise from the same sources as in the main experiment and were processed in the same way. In contrast to the main experiment we did not restrict our analysis to the temperate zone of the European and North American continents. To minimize the confounding effect of potential differences in topographic heterogeneity between the continents, we excluded all grid cells with an elevation greater than 1000 m from subsequent analyses (Figure 1 and Figure 2).


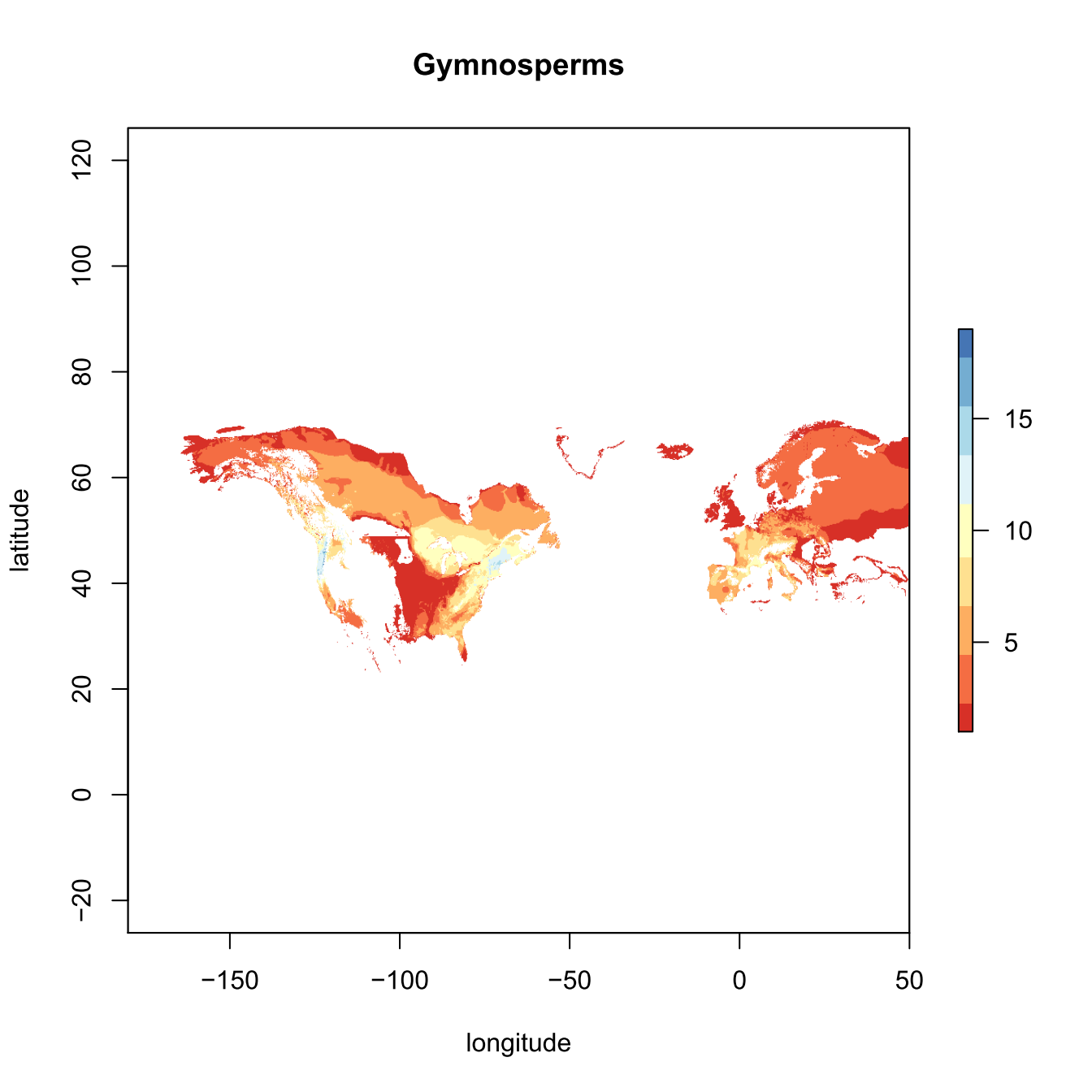


Figure 1 Species richness map for 94 Gymnosperms.


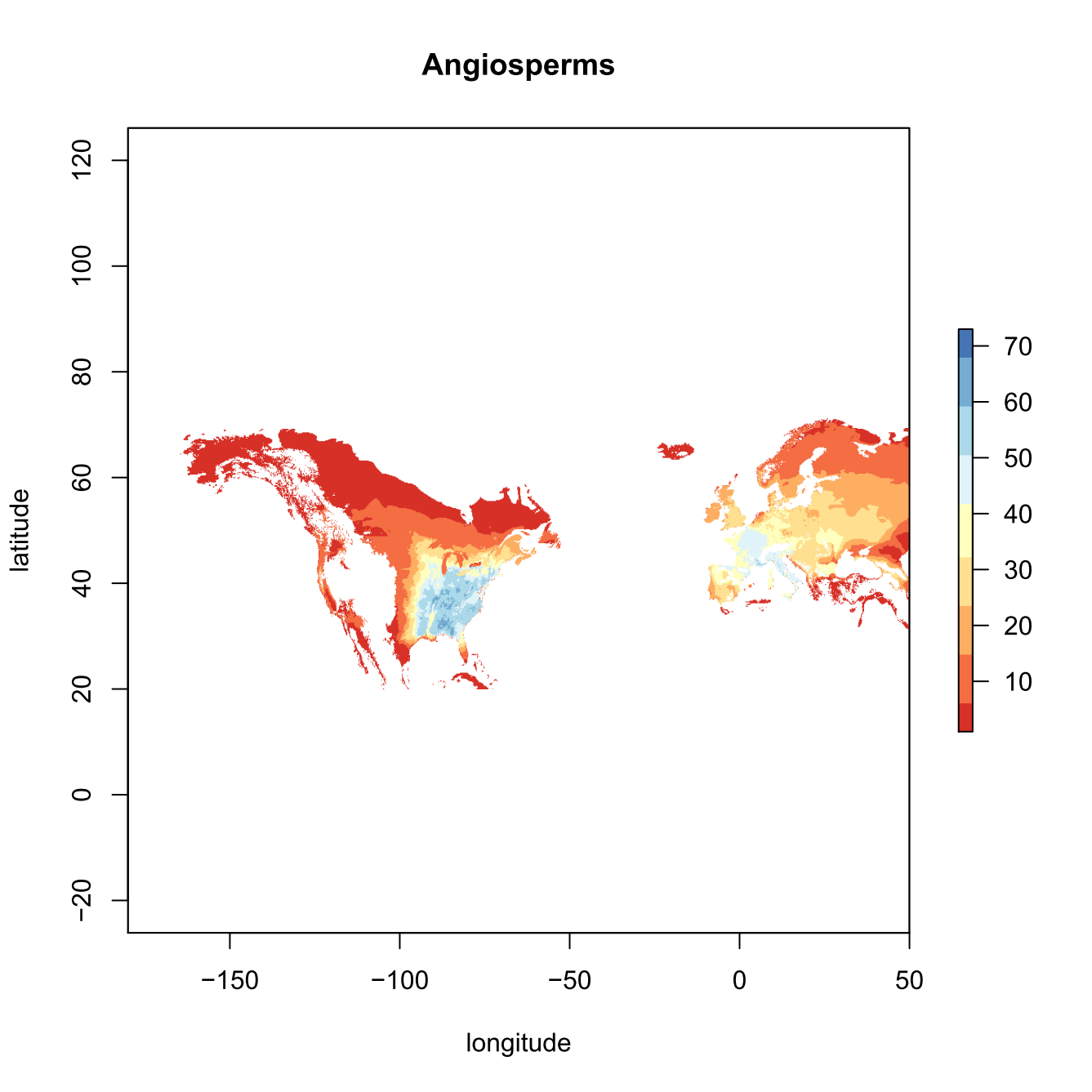


Figure 2 Species richness map for 227 Angiosperms.

1.3. Statistical analyses

All analyses were performed in the same way as in the main experiment.

2. RESULTS

*Gymnosperms*

For the gymnosperms, the functional dispersion of species in trait space is not different between Europe and North America. This pattern is consistent for the continental as well as the grid-cell scale (Figure 3, Figure 4, Figure 5, Figure 6). This means that neither in the location of the centroid nor in the dispersion of species in trait space a significant difference between the continents can be detected (Table 1). Thus, the results are in accordance with the results of the main experiment.

*Angiosperms*

For the angiosperms we find significant differences in trait space occupation between North America and Europe at the continental and the grid-cell scale (Figure 3, Figure 4, Figure 5, Figure 6). At the grid-cell scale we find a rapid increase of FDis with species richness in all North American subregions (Figure 3) peaking at intermediate species richness levels and subsequently declining. While at low species richness levels this pattern is ambiguous, European tree assemblages at higher species richness levels consistently exhibit a greater FDis. This result is consistent with the findings at the continental scale, where the dispersion of European tree species is significantly greater than the dispersion of North American species (Table 1). Moreover, the results are in accordance with the results of the main experiment.


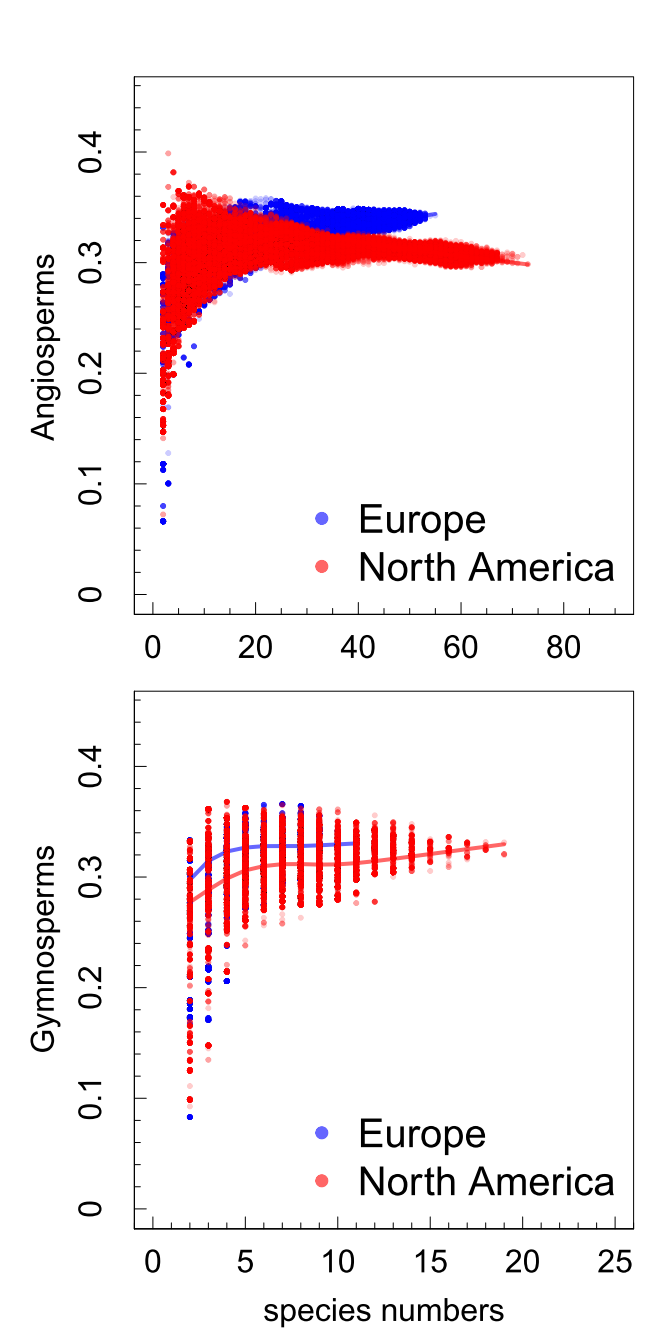


Figure 3 The relationship of species richness and functional dispersion for European and North American gymnosperm and angiosperm communities, based on 5arcmin species distribution maps.


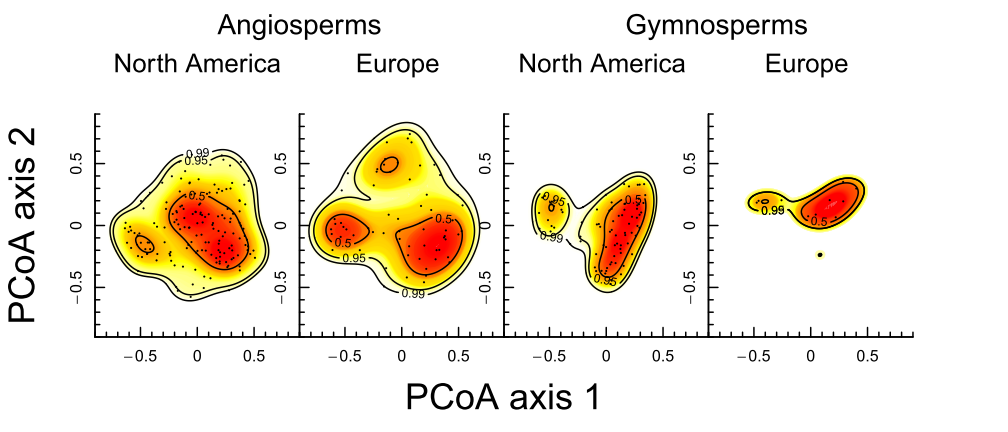


Figure 4 Kernel density estimations of the first two PCoA-axes based on a Gower distance matrix of 26 traits for 164 North American and 63 European angiosperms, as well as for 82 North American and 12 European gymnosperms.


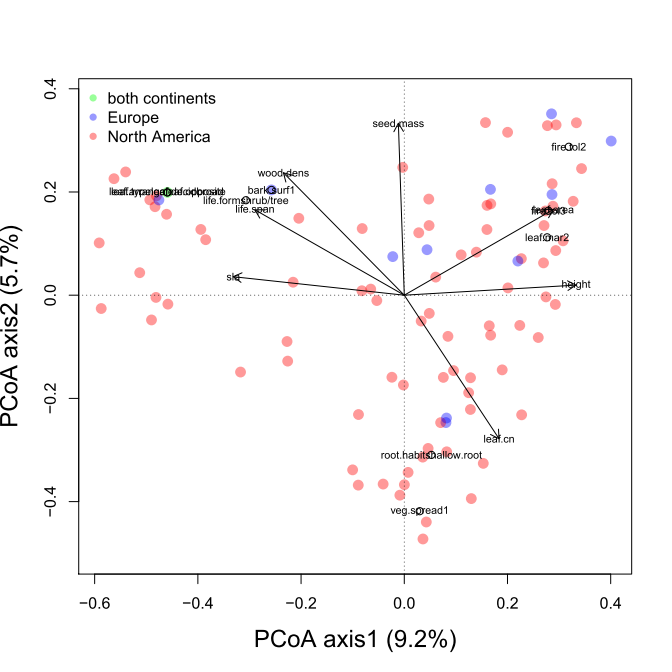


Figure 5 PCoA ordination plot showing distances among North American and European woody gymnosperm species based on 22 traits for the first two axes. Correlations with a loading of min +/- 0.25 of traits on the first two PCoA axes are represented as arrows; the lengths of the arrows are proportional to their correlation coefficient, and they point in the direction of most rapid change; nominal traits were dummy coded before correlation.


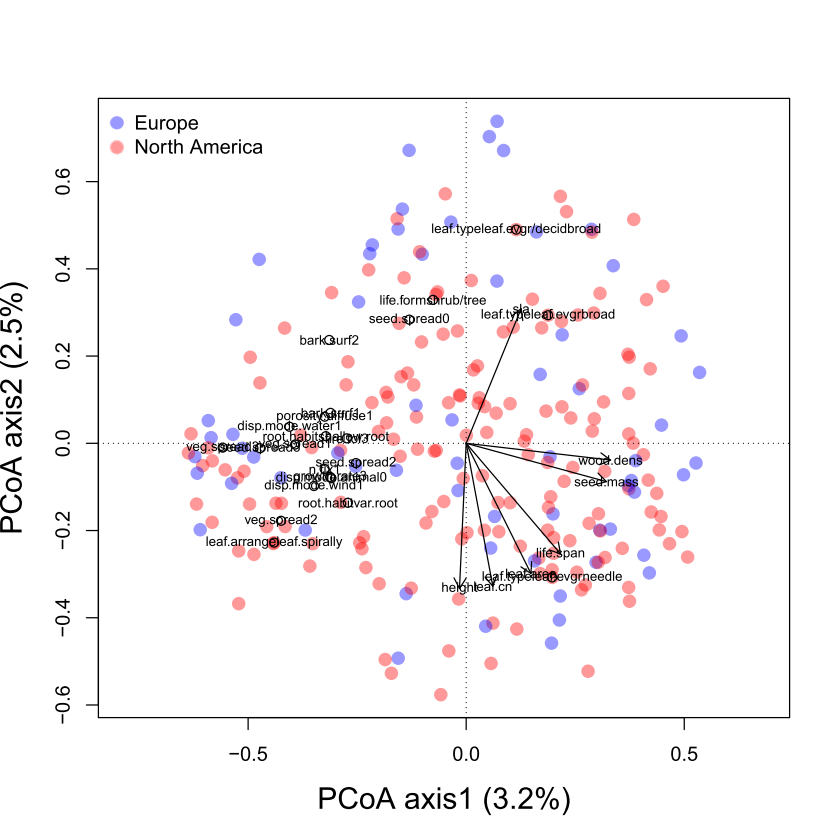


Figure 6 PCoA ordination plot showing distances among North American and European woody angiosperm species based on 26 traits for the first two axes. Correlations with a loading of min +/- 0.25 of traits on the first two PCoA axes are represented as arrows; the lengths of the arrows are proportional to their correlation coefficient, and they point in the direction of most rapid change; nominal traits were dummy coded before correlation.

Table 1 Summary of the permutation test for differences in multivariate homogeneity of group dispersions (Functional dispersion) between the continents based on 999 permutations, and the perMANOVA for differences in variance between the functional clouds based

|  |  |  | ***multivariate homogeneity of group dispersions (FDis)*** | | ***variance between the functional clouds (perMANOVA)*** | |
| --- | --- | --- | --- | --- | --- | --- |
| **class** | **region** | **df** | ***F*** | ***p.val*** | ***pseudo-F*** | ***p.val*** |
| gymnosperms | Whole continent | 1 | 2.0112 | 0.177 | 1.5732 | 0.093 |
| angiosperms | **Whole continent** | **1** | **11.001** | **0.002** | **8.2019** | **<0.001** |

3. DISCUSSION

The analysis undertaken on whole continental scale without any climatic restriction and with 100 additional tree species confirm the results of the main experiment. This strongly suggests that our findings are driven by a robust connection between the functional diversity and species richness on both continents.
