# Supplementary material for: Functional Resilience against Climate-Driven Extinctions – Comparing the Functional Diversity of European and North American Tree Floras
Source: PLoS One. 2016 Feb 5;11(2):e0148607. doi: 10.1371/journal.pone.0148607 (PMC4743854; doi:10.1371/journal.pone.0148607)
Supplement: S4 File — (DOCX) [file pone.0148607.s004.docx]

# Appendix S4 File - Analysis undertaken for temperate zones according to the Koeppen-Geiger climate classification

1. MATERIALS AND METHODS

1.1. Trait data

We assembled a trait matrix for 26 traits including 229 North American (146 angiosperms, 63 gymnosperms) and 74 European tree species (63 angiosperms, 11 gymnosperms) from the whole continent. A single species, *Juniperus communis*, occurred on both continents. The species were chosen according to the same criteria as in the main experiment.

In addition to the species set from the main experiment the following 26 Gymnosperms were included in the analyses: *Abies amabilis, Abies grandis, Abies lasiocarpa, Abies procera, Chamaecyparis nootkatensis, Juniperus monosperma, Juniperus occidentalis, Juniperus pinchotii, Juniperus scopulorum, Larix kaempferi, Larix occidentalis, Picea engelmannii, Picea sitchensis, Pinus albicaulis, Pinus cembroides, Pinus clausa, Pinus contorta, Pinus monticola, Pinus ponderosa, Pinus radiata, Pseudotsuga menziesii, Taxus brevifolia, Thuja plicata, Torreya taxifolia, Tsuga heterophylla, Tsuga mertensiana*.

The following 20 Angiosperms were included in the analyses: *Acer glabrum, Acer grandidentatum, Acer macrophyllum, Alnus rhombifolia, Alnus rubra, Arbutus menziesii, Betula occidentalis, Cercocarpus ledifolius, Cornus nuttallii, Cydonia oblonga, Platanus orientalis, Populus trichocarpa, Prunus emarginata, Punica granatum, Quercus faginea, Quercus garryana, Quercus macranthera, Quercus pontica, Rhizophora mangle, Syringa vulgaris.* Due the new spatial subset Quercus ellipsoidalis which was used in the main experiment was not present in the new dataset.

1.2. Spatial data

All species distribution maps arise from the same sources as in the main experiment and were processed in the same way. In contrast to the main experiment we did not restrict our analysis to the temperate zone of the European and North American continents. To minimize the confounding effect of potential differences in topographic heterogeneity between the continents, we excluded all grid cells with an elevation greater than 1000 m from subsequent analyses (Figure 1 and Figure 2). We restricted the analyzed area on the European and North American continents according to the Koeppen-Geiger climate classification zones "Cfa" and "Cfb" (Kottek et al., 2006; Peel et al., 2007). Zone "Cf" specifies temperate climate (temperature of the hottest month > 10°C & 0°C < temperature of the coldest month <18°C) without a dry season. "Cfa" specifies areas with hot summers (temperature of the hottest month ≥ 22). Within this general climate zone "Cfb" relates to areas with warm summers (Not "Cfa" & number of months where the temperature is above 10 ≥ 4).


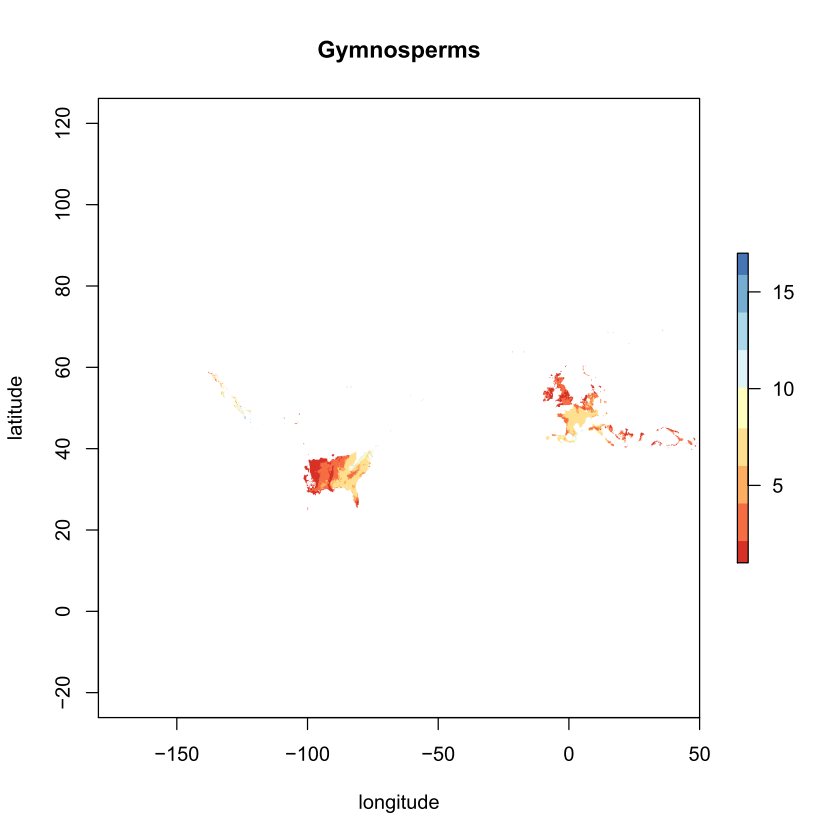


Figure 1 Species richness map for 75 Gymnosperms.


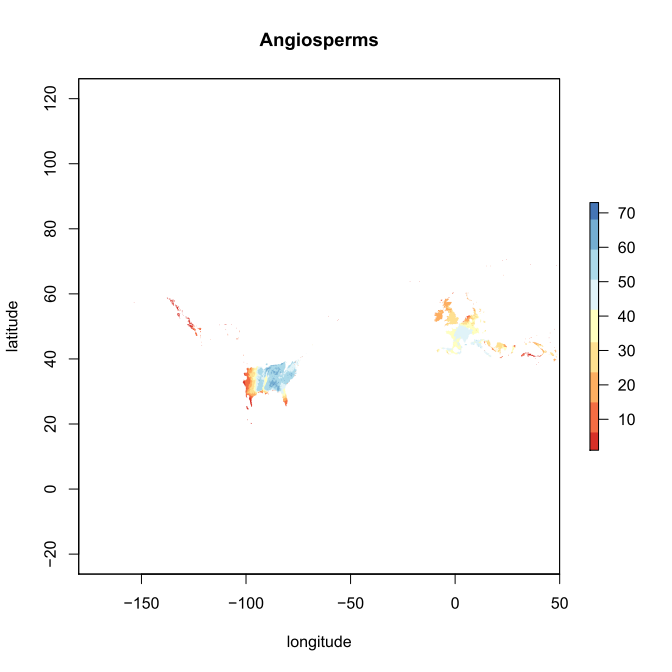


Figure 2 Species richness map for 209 Angiosperms.

1.3. Statistical analyses

All analyses were performed in the same way as in the main experiment.

2. RESULTS

*Gymnosperms*

For the gymnosperms, the functional dispersion of species in trait space is not different between Europe and North America. This pattern is consistent for the continental as well as the grid-cell scale (Figure 3, Figure 4, Figure 5, Figure 6). This means that neither in the location of the centroid nor in the dispersion of species in trait space a significant difference between the continents can be detected (Table 1). Thus, the results are in accordance with the results of the main experiment.

*Angiosperms*

For the angiosperms we find significant differences in trait space occupation between North America and Europe at the continental and the grid-cell scale (Figure 3, Figure 4, Figure 5, Figure 6). At the grid-cell scale we find a rapid increase of FDis with species richness in all North American subregions (Figure 3) peaking at intermediate species richness levels and subsequently declining. While at low species richness levels this pattern is ambiguous, European tree assemblages at higher species richness levels consistently exhibit a greater FDis. This result is consistent with the findings at the continental scale, where the dispersion of European tree species is significantly greater than the dispersion of North American species (Table 1). Moreover, the results are in accordance with the results of the main experiment.


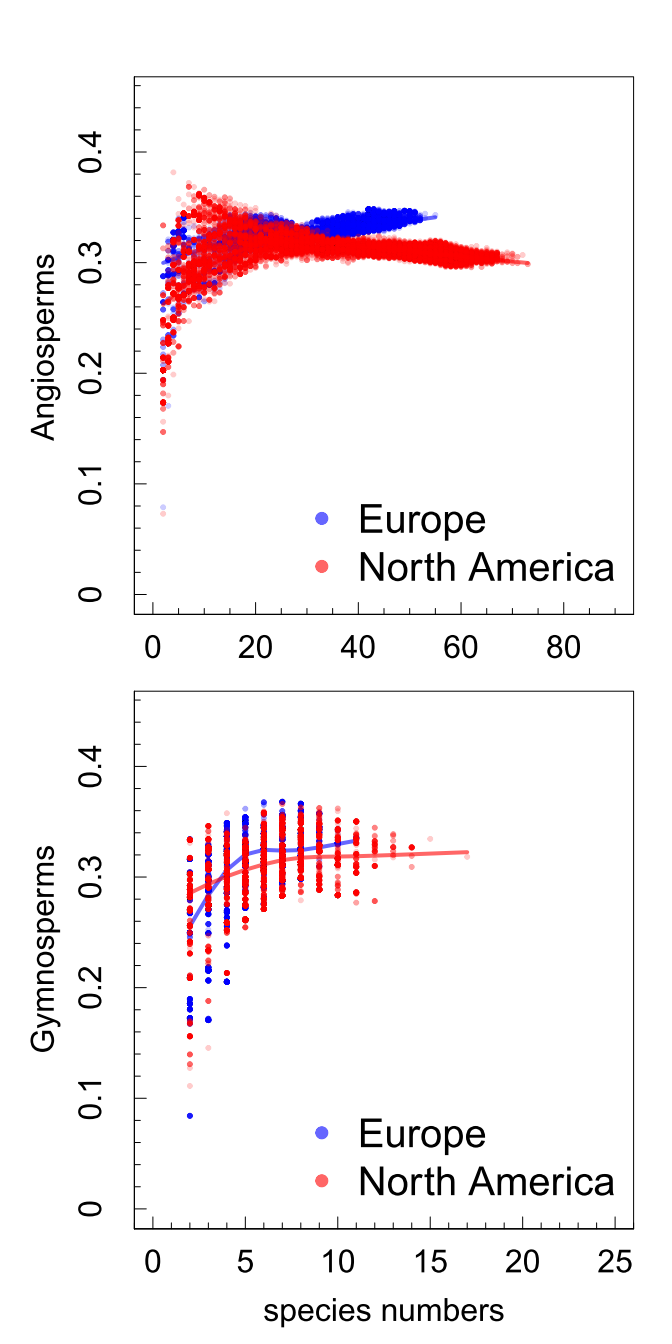


Figure 3 The relationship of species richness and functional dispersion for European and North American gymnosperm and angiosperm communities, based on 5arcmin species distribution maps.


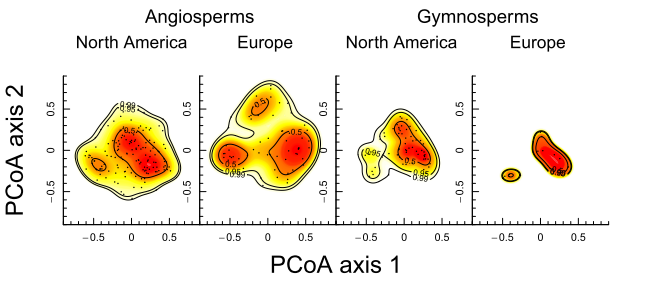


Figure 4 Kernel density estimations of the first two PCoA-axes based on a Gower distance matrix of 26 traits for 146 North American and 63 European angiosperms, as well as for 64 North American and 12 European gymnosperms.


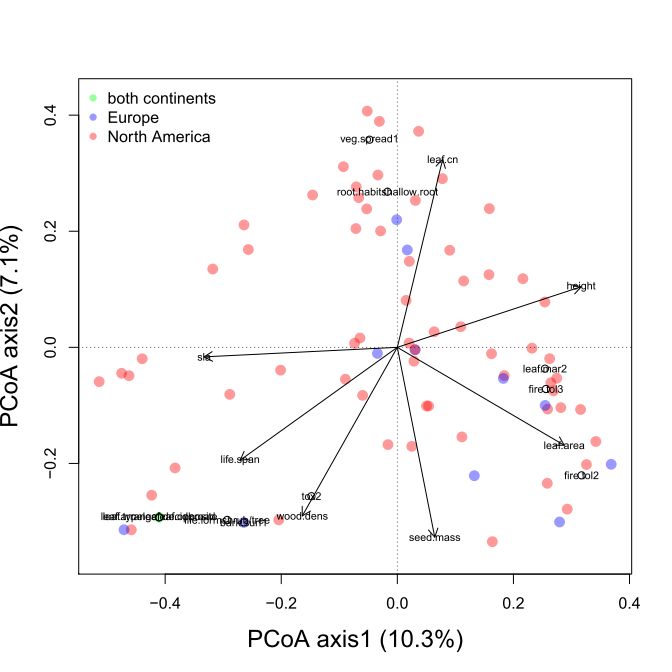


Figure 6 PCoA ordination plot showing distances among North American and European woody gymnosperm species based on 22 traits for the first two axes. Correlations with a loading of min +/- 0.25 of traits on the first two PCoA axes are represented as arrows; the lengths of the arrows are proportional to their correlation coefficient, and they point in the direction of most rapid change; nominal traits were dummy coded before correlation.


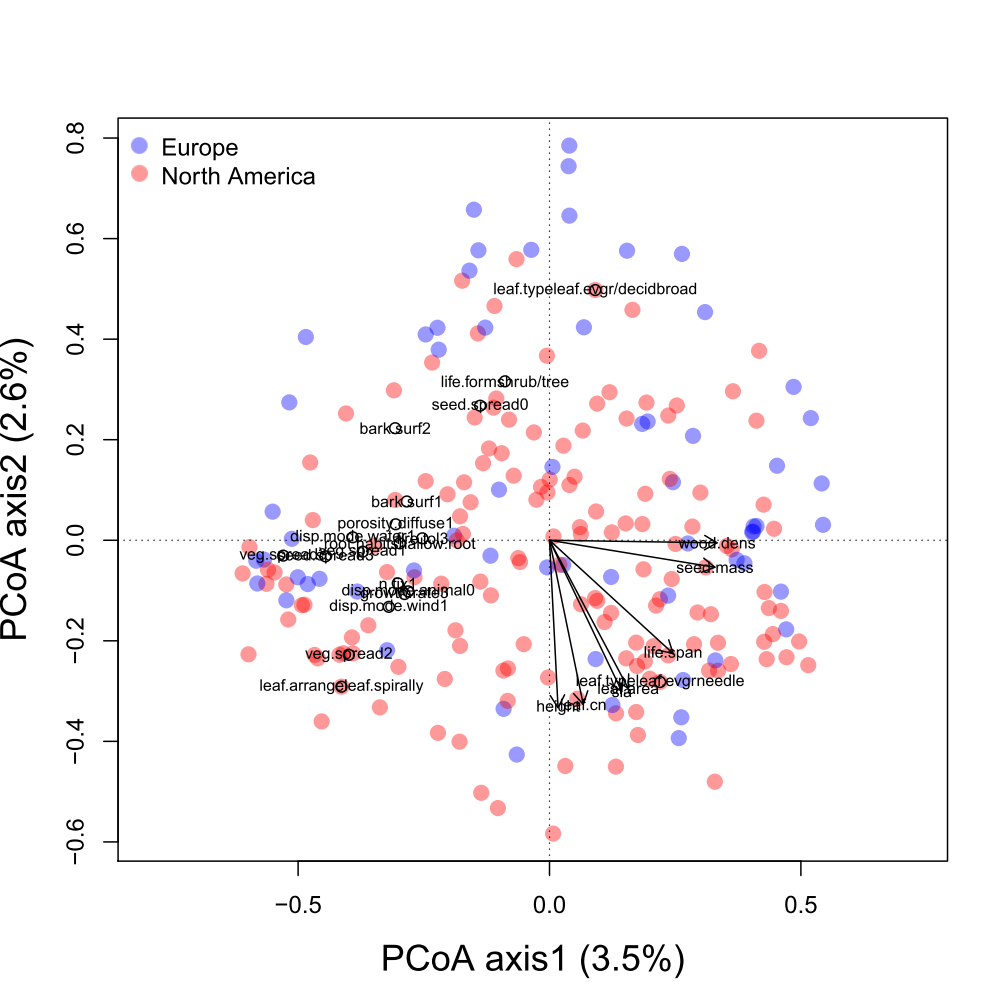


Figure 7 PCoA ordination plot showing distances among North American and European woody angiosperm species based on 26 traits for the first two axes. Correlations with a loading of min +/- 0.25 of traits on the first two PCoA axes are represented as arrows; the lengths of the arrows are proportional to their correlation coefficient, and they point in the direction of most rapid change; nominal traits were dummy coded before correlation.

Table 1 Summary of the permutation test for differences in multivariate homogeneity of group dispersions (Functional dispersion) between the continents based on 999 permutations, and the perMANOVA for differences in variance between the functional clouds based

|  |  |  | ***multivariate homogeneity of group dispersions (FDis)*** | | ***variance between the functional clouds (perMANOVA)*** | |
| --- | --- | --- | --- | --- | --- | --- |
| **class** | **region** | **df** | ***F*** | ***p.val*** | ***pseudo-F*** | ***p.val*** |
| gymnosperms | Whole continent | 1 | 1.1505 | 0.279 | 1.4418 | 0.1382 |
| angiosperms | **Whole continent** | **1** | **11.375** | **0.001** | **7.9206** | **<0.001** |

3. DISCUSSION

The analysis for the Koeppen-Geiger climate classification zones "Cfa" and "Cfb" with 46 additional tree species confirm the results of the main experiment. This strongly suggests that our findings are driven by a robust connection between the functional diversity and species richness on both continents.

4.REFERENCES

Kottek M., Grieser J., Beck C., Rudolf B., & Rubel F. (2006) World Map of the Köppen-Geiger climate classification updated. Meteorologische Zeitschrift, 15, 259–263.

Peel M.C., Finlayson B.L., & Mcmahon T.A. (2007) Updated world map of the Koeppen-Geiger climate classification map. Hydrology and Earth System Sciences, 11, 1633–1644.
