# Supplementary material for: Functional Resilience against Climate-Driven Extinctions – Comparing the Functional Diversity of European and North American Tree Floras
Source: PLoS One. 2016 Feb 5;11(2):e0148607. doi: 10.1371/journal.pone.0148607 (PMC4743854; doi:10.1371/journal.pone.0148607)
Supplement: S5 File — (DOCX) [file pone.0148607.s005.docx]

# Appendix S5 File - Functional Richness analyses

Statistical analysis

All analyses for FDis were also performed with functional richness (FRic). Distance matrices from step 1 (main text) were used to calculate functional diversity indices at the continental scale, at the sub-regional scale (i.e. considering the four climatically similar regions described in Section 2.2) and at the grid-scale with a 5 arc min resolution. We calculated the functional diversity index Functional richness (FRic, Villéger et al., 2008) which measures the volume of the n-dimensional trait space. Prior to the FRic calculation a Principle coordinate analysis (PCoA) was necessary to transform the distance matrix into rescaled trait values (Laliberté & Legendre 2010). Because FRic calculation is problematic for high-dimensional data we used different numbers of Principle components to calculate FRic at different scales: At the grid-cell scale the calculation of FRic is based on the first two PCoA-axes (for reasons of computational feasability). To correct for negative eigenvalues of PCoA-axes, we used Cailliez correction (FD package in R; Laliberté, Legendre, & Shipley, 2014). At the scale of the sub-regions and the continents we calculated FRic for 2 dimensions as well as for ten dimensions to ensure the reliability of the results obtained for 2 dimensions. FRic was calculated using the convex hull algorithm (geometry package in R; Barber et al., 2014). Due to the larger numbers of species in North America the number of axes needed to explain the whole variance in the PCoAs was also larger in North America. To make European and North American FRic comparable we standardized the convex hulls by the axes needed to explain the variation on each continent.

Results

*Gymnosperms*

For the Gymnosperms, the functional richness of species in trait space is not different between Europe and North America (Figure 1, Table 1). FRic increases monotonically with increasing species numbers on both continents but due to higher total numbers of North American gymnosperms the maximum functional richness reached on the continent is higher than in Europe. This is supported by the numerical analyses of FRic: neither the convex hull based on two nor on ten is different between the continents for gymnosperms.

*Angiosperms*

In contrast, angiosperms differ in functional richness of species between North America and Europe on the continental, the sub-regional and the grid-cell scale (Figure 2, Table 1). The association patterns of grid-cell based FRic to species richness in North America reveal a monotonic increment with species number, but the increment in North America is slower, leading to visibly lower FRic at high species numbers in North America than in Europe. Accordingly the 2- and 10-dimensional volume of the convex hulls are different between the continents for angiosperms.

Discussion

Our findings show that a higher climatically driven loss of tree species in Europe as compared to North America during the last glaciations did not led to a reduced functional diversity. While the functional richness among gymnosperms is comparable for both continents the functional richness of angiosperms in consistently greater in Europe.

Figures and tables


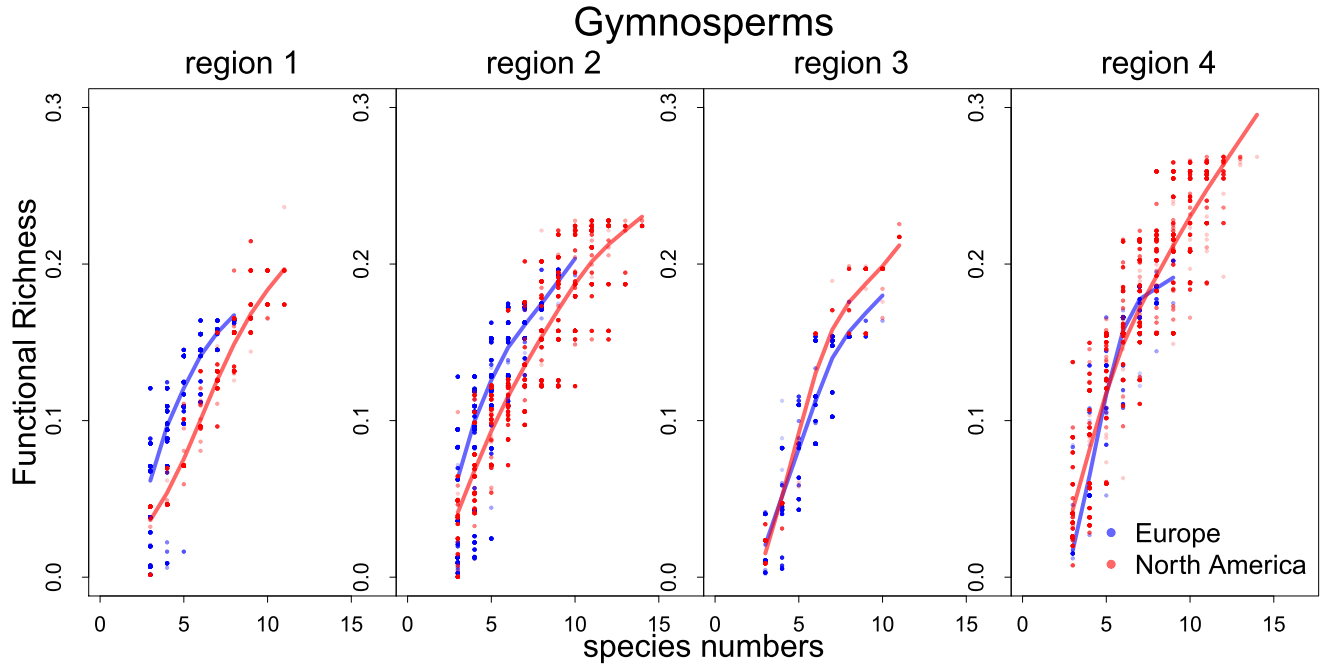


Figure S2: The relationship of species richness and functional richness for European and North American gymnosperm communities, based on 5arcmin species distribution maps.


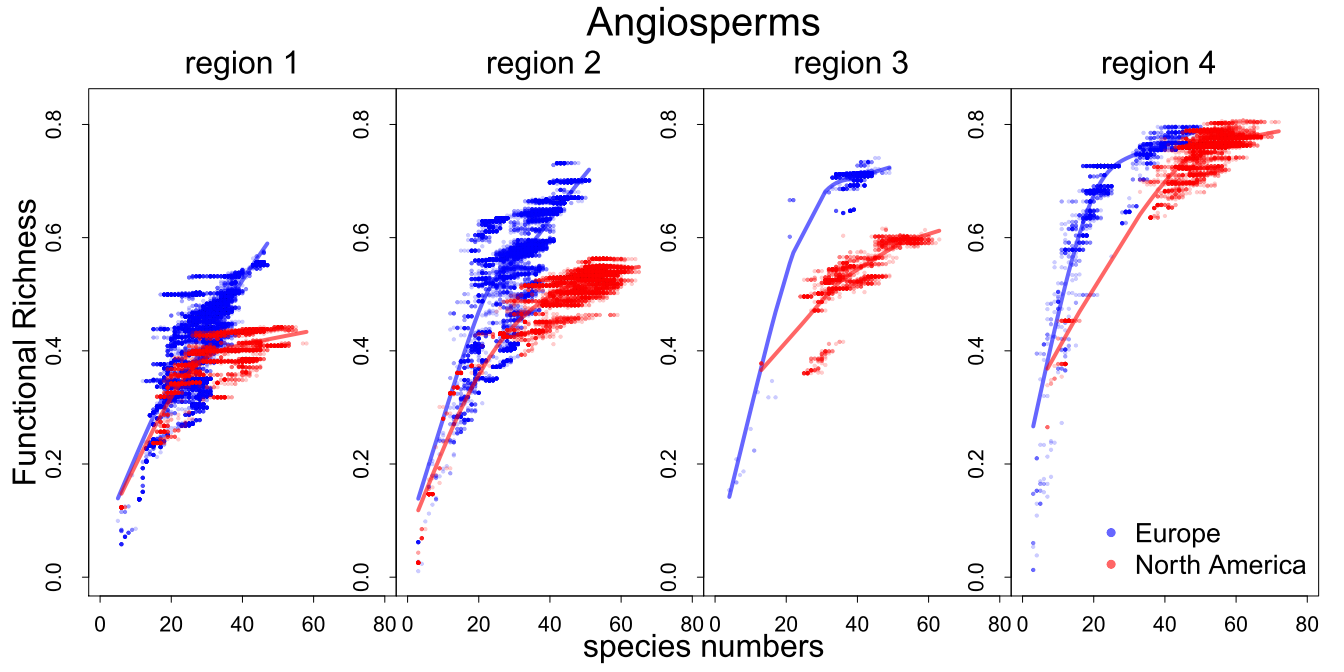


Figure S3: The relationship of species richness and functional richness for European and North American angiosperm communities, based on 5arcmin species distribution maps.

Table S5: Size of the convex hulls for different regions and numbers of axes.

|  |  | Angiosperms | | Gymnosperms | |
| --- | --- | --- | --- | --- | --- |
|  |  | North America | Europe | North America | Europe |
| region 1 | axes needed to represent the whole variation | 43 | 30 | 14 | 10 |
|  | convex hull volume of 2 axes | 2.13E-03 | 3.63E-03 | 1.33E-02 | 1.97E-02 |
|  | convex hull volume of 10 axes | 1.17E-10 | 1.74E-10 | 8.00E-12 | 2.44E-13 |
|  |  |  |  |  |  |
| region 2 | axes needed to represent the whole variation | 50 | 30 | 19 | 10 |
|  | convex hull volume of 2 axes | 1.56E-03 | 3.98E-03 | 9.03E-03 | 1.97E-02 |
|  | convex hull volume of 10 axes | 1.42E-10 | 1.86E-10 | 3.47E-11 | 2.44E-13 |
|  |  |  |  |  |  |
| region 3 | axes needed to represent the whole variation | 53 | 31 | 20 | 10 |
|  | convex hull volume of 2 axes | 1.43E-03 | 4.01E-03 | 7.82E-03 | 1.97E-02 |
|  | convex hull volume of 10 axes | 1.41E-10 | 1.89E-10 | 3.17E-11 | 2.44E-13 |
|  |  |  |  |  |  |
| region 4 | axes needed to represent the whole variation | 54 | 31 | 22 | 10 |
|  | convex hull volume of 2 axes | 1.39E-03 | 4.14E-03 | 6.67E-03 | 1.97E-02 |
|  | convex hull volume of 10 axes | 1.36E-10 | 1.62E-10 | 4.62E-11 | 2.44E-13 |

References

Barber, C.B. et al., 2014. Mesh generation and surface tesselation, R package “geometry”, Version 0.3-4.

Laliberté, E. & Legendre, P., 2010. A distance-based framework for measuring functional diversity from multiple traits. *Ecology*, 91(1), pp.299–305. Available at: http://www.ncbi.nlm.nih.gov/pubmed/20380219.

Laliberté, E., Legendre, P. & Shipley, B., 2014. Measuring functional diversity (FD) from multiple traits, and other tools for functional ecology, R package “FD” Version 1.0-12.

Villéger, S., Mason, N.W.H. & Mouillot, D., 2008. New multidimensional functional diversity indices for a multifaceted framework in functional ecology. *Ecology*, 89(8), pp.2290–301. Available at: http://www.ncbi.nlm.nih.gov/pubmed/18724739.
