# Supplementary material for: Functional Resilience against Climate-Driven Extinctions – Comparing the Functional Diversity of European and North American Tree Floras
Source: PLoS One. 2016 Feb 5;11(2):e0148607. doi: 10.1371/journal.pone.0148607 (PMC4743854; doi:10.1371/journal.pone.0148607)
Supplement: S6 File — (DOCX) [file pone.0148607.s006.docx]

# Appendix S6 File - Additional information for the PCoA - analyses


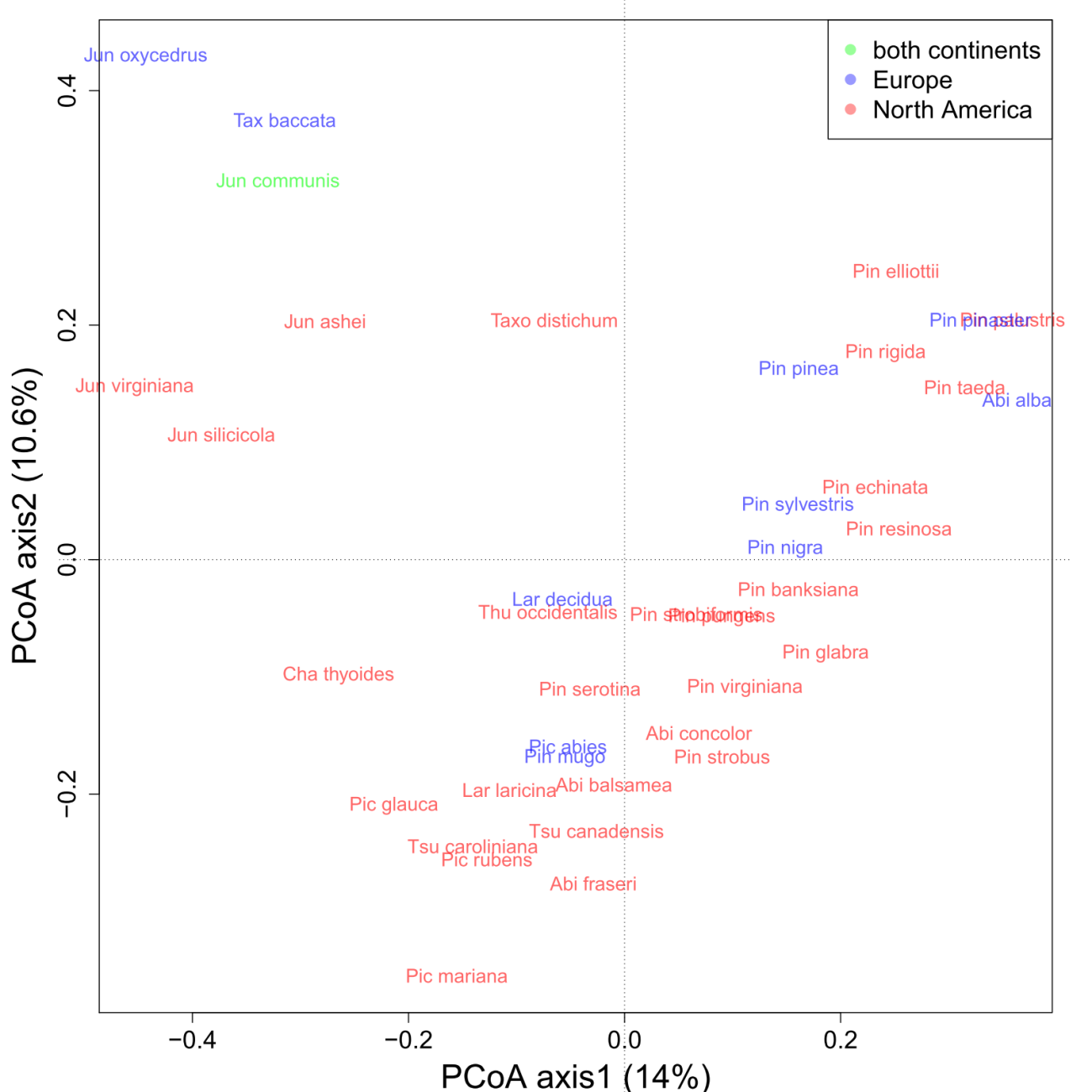


Figure 1: PCoA ordination plot showing distances among 28 North American and 10 European woody gymnosperm species based on 22 traits for the first two axes. Genus abbreviations: Abi: Abies, Cha: Chamaecyparis, Jun: Juniperus, Lar: Larix, Pic: Picea, Pin: Pinus, Taxo: Taxodium, Tax: Taxus, Thu: Thuja, Tsu: Tsuga,


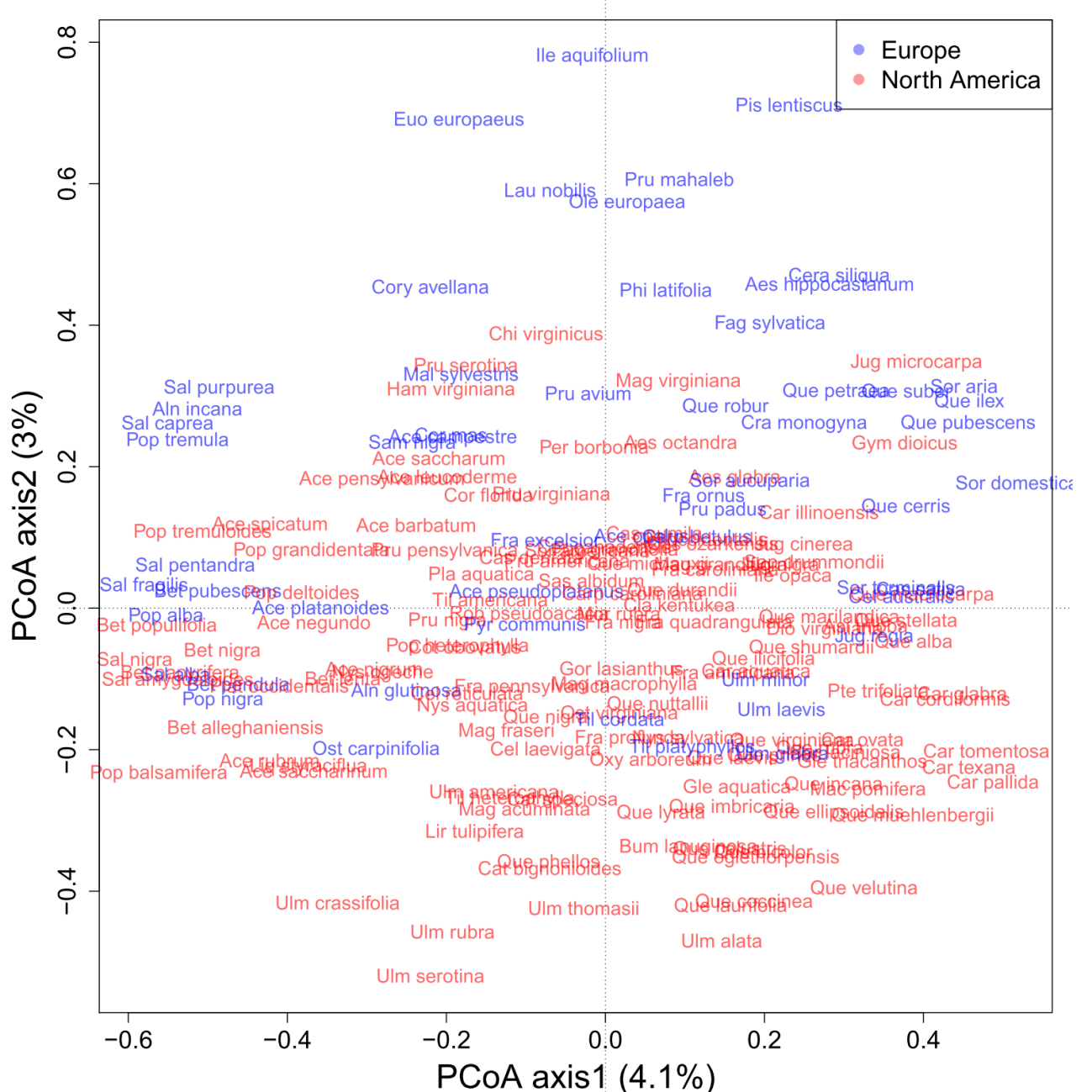


Figure 2: PCoA ordination plot showing distances among 126 North American and 56 European woody angiosperm species based on 26 traits for the first two axes. Genus abbreviation: Ace: Acer, Aes: Aesculus, Aln: Alnus, Asi: Asimina, Bet: Betula, Bum: Bumelia, Carp: Carpinus, Car: Carya, Cas: Castanea, Cat: Catalpa, Cel: Celtis, Cera: Ceratonia, Cer: Cercis, Chi: Chionanthus, Cla: Cladrastis, Cor: Cornus, Cory: Corylus, Cot: Cotinus, Cra: Crataegus, Dio: Diospyros, Euo: Euonymus, Fag: Fagus, Fra: Fraxinus, Gle: Gleditsia, Gor: Gordonia, Gym: Gymnocladus, Ham: Hamamelis, Ile: Ilex, Jug: Juglans, Lau: Laurus, Liq: Liquidambar, Lir: Liriodendron, Mac: Maclura, Mag: Magnolia, Mal: Malus, Mor: Morus, Nys: Nyssa, Ole: Olea, Ost: Ostrya, Oxy: Oxydendrum, Per: Persea, Phi: Phillyrea, Pis: Pistacia, Pla: Planera, Plat: Platanus, Pop: Populus, Pru: Prunus, Pte: Ptelea, Pyr: Pyrus, Que: Quercus, Rob: Robinia, Sal: Salix, Sam: Sambucus, Sap: Sapindus, Sas: Sassafras, Sor: Sorbus, Til: Tilia, Ulm: Ulmus

Table 1: Cumulative explained variance of the first 10 PCoA-axes.

| axis | explained variance [%] | |
| --- | --- | --- |
|  | angiosperms | gymnosperms |
| 1 | 4.0 | 14.0 |
| 2 | 7.0 | 24.6 |
| 3 | 9.9 | 32.3 |
| 4 | 12.3 | 39.0 |
| 5 | 14.5 | 44.4 |
| 6 | 16.5 | 49.2 |
| 7 | 18.2 | 53.2 |
| 8 | 19.8 | 57.0 |
| 9 | 21.3 | 60.2 |
| 10 | 22.8 | 63.3 |
