# Supplementary material for: Functional Resilience against Climate-Driven Extinctions – Comparing the Functional Diversity of European and North American Tree Floras
Source: PLoS One. 2016 Feb 5;11(2):e0148607. doi: 10.1371/journal.pone.0148607 (PMC4743854; doi:10.1371/journal.pone.0148607)
Supplement: S8 File — (DOCX) [file pone.0148607.s008.docx]

Dear Mario,

I grant permission to the open-access journal PLOS ONE to publish an

image created on the basis of a worldclim.org-dataset under the

Creative Commons Attribution License (CCAL) CC BY 4.0

(<http://creativecommons.org/licenses/by/4.0/>).

I am aware that this license allows unrestricted use and distribution,

even commercially, by third parties.

Best regards, Robert Hijmans
