# Supplementary material for: Functional Resilience against Climate-Driven Extinctions – Comparing the Functional Diversity of European and North American Tree Floras
Source: PLoS One. 2016 Feb 5;11(2):e0148607. doi: 10.1371/journal.pone.0148607 (PMC4743854; doi:10.1371/journal.pone.0148607)
Supplement: S1 Table — (DOCX) [file pone.0148607.s009.docx]

# Appendix S1 Table - Species sample

| taxa names used for the analysis | comments | accepted taxa names |
| --- | --- | --- |
| *Abies alba* |  | *Abies alba Mill.* |
| *Abies balsamea* |  | *Abies balsamea (L.) Mill.* |
| *Abies concolor* |  | *Abies concolor (Gordon) Lindl. ex Hildebr.* |
| *Abies fraseri* |  | *Abies fraseri (Pursh) Poir.* |
| *Acer barbatum* | synonym not resolvable without specific authority | *Acer (barbatum)* |
| *Acer campestre* |  | *Acer campestre L.* |
| *Acer leucoderme* | synonym | *Acer saccharum subsp. leucoderme (Small) Desmarais* |
| *Acer negundo* |  | *Acer negundo L.* |
| *Acer nigrum* | synonym | *Acer saccharum subsp. nigrum (F.Michx.) Desmarais* |
| *Acer opalus* |  | *Acer opalus Mill.* |
| *Acer pensylvanicum* |  | *Acer pensylvanicum L.* |
| *Acer platanoides* |  | *Acer platanoides L.* |
| *Acer pseudoplatanus* |  | *Acer pseudoplatanus L.* |
| *Acer rubrum* |  | *Acer rubrum L.* |
| *Acer saccharinum* |  | *Acer saccharinum L.* |
| *Acer saccharum* |  | *Acer saccharum Marshall* |
| *Acer spicatum* |  | *Acer spicatum Lam.* |
| *Aesculus glabra* |  | *Aesculus glabra Willd.* |
| *Aesculus hippocastanum* |  | *Aesculus hippocastanum L.* |
| *Aesculus octandra* | synonym | *Aesculus flava Sol.* |
| *Alnus glutinosa* |  | *Alnus glutinosa (L.) Gaertn.* |
| *Alnus incana* |  | *Alnus incana (L.) Moench* |
| *Asimina triloba* |  | *Asimina triloba (L.) Dunal* |
| *Betula alleghaniensis* |  | *Betula alleghaniensis* |
| *Betula lenta* |  | *Betula lenta L.* |
| *Betula nigra* |  | *Betula nigra L.* |
| *Betula papyrifera* |  | *Betula papyrifera Marshall* |
| *Betula pendula* |  | *Betula pendula Roth* |
| *Betula populifolia* |  | *Betula populifolia Marshall* |
| *Betula pubescens* |  | *Betula pubescens Ehrh.* |
| *Bumelia lanuginosa* | synonym | *Sideroxylon lanuginosum Michx.* |
| *Carpinus betulus* |  | *Carpinus betulus L.* |
| *Carpinus caroliniana* |  | *Carpinus caroliniana Walter* |
| *Carya aquatica* |  | *Carya aquatica (F.Michx.) Nutt. ex Elliott* |
| *Carya cordiformis* |  | *Carya cordiformis (Wangenh.) K.Koch* |
| *Carya glabra* |  | *Carya glabra (Mill.) Sweet* |
| *Carya illinoensis* |  | *Carya illinoensis K.Koch* |
| *Carya laciniosa* |  | *Carya laciniosa (F. Michx.) Loudon* |
| *Carya ovata* |  | *Carya ovata (Mill.) K.Koch* |
| *Carya pallida* |  | *Carya pallida (Ashe) Engelm. & Graebn.* |
| *Carya texana* |  | *Carya texana Buckley* |
| *Carya tomentosa* | synonym | *Carya alba (L.) Nutt. ex Elliott* |
| *Castanea dentata* |  | *Castanea dentata (Marshall) Borkh.* |
| *Castanea ozarkensis* |  | *Castanea ozarkensis Ashe* |
| *Castanea pumila* |  | *Castanea pumila (L.) Mill.* |
| *Castanea sativa* |  | *Castanea sativa Mill* |
| *Catalpa bignonioides* |  | *Catalpa bignonioides Walter* |
| *Catalpa speciosa* |  | *Catalpa speciosa (Warder ex Barney) Warder ex Engelm.* |
| *Celtis australis* |  | *Celtis australis L.* |
| *Celtis laevigata* |  | *Celtis laevigata Willd.* |
| *Celtis occidentalis* |  | *Celtis occidentalis L.* |
| *Celtis reticulata* | synonym | *Celtis laevigata var. reticulata (Torr.) Benson* |
| *Ceratonia siliqua* |  | *Ceratonia siliqua L.* |
| *Cercis canadensis* |  | *Cercis canadensis L.* |
| *Chamaecyparis thyoides* |  | *Chamaecyparis thyoides (L.) Britton, Sterns & Poggenb.* |
| *Chionanthus virginicus* |  | *Chionanthus virginicus L.* |
| *Cladrastis kentukea* |  | *ladrastis kentukea (Dum. Cours.) Rudd* |
| *Cornus florida* |  | *Cornus florida L.* |
| *Cornus mas* |  | *Cornus mas L.* |
| *Corylus avellana* |  | *Corylus avellana L.* |
| *Cotinus obovatus* |  | *Cotinus obovatus Raf.* |
| *Crataegus monogyna* |  | *Crataegus monogyna Jacq.* |
| *Diospyros virginiana* |  | *Diospyros virginiana L.* |
| *Euonymus europaeus* |  | *Euonymus europaeus L.* |
| *Fagus grandifolia* |  | *Fagus grandifolia Ehrh.* |
| *Fagus sylvatica* |  | *Fagus sylvatica L.* |
| *Fraxinus americana* |  | *Fraxinus americana L.* |
| *Fraxinus caroliniana* |  | *Fraxinus caroliniana Mill.* |
| *Fraxinus excelsior* |  | *Fraxinus excelsior L.* |
| *Fraxinus nigra* |  | *Fraxinus nigra Marshall* |
| *Fraxinus ornus* |  | *Fraxinus ornus L.* |
| *Fraxinus pennsylvanica* |  | *Fraxinus pennsylvanica Marshall* |
| *Fraxinus profunda* |  | *Fraxinus profunda (Bush) Bush* |
| *Fraxinus quadrangulata* |  | *Fraxinus quadrangulata Michx.* |
| *Gleditsia aquatica* |  | *Gleditsia aquatica Marshall* |
| *Gleditsia triacanthos* |  | *Gleditsia triacanthos L.* |
| *Gordonia lasianthus* |  | *Gordonia lasianthus (L.) J. Ellis* |
| *Gymnocladus dioicus* | synonym (spelling variant) | *Gymnocladus dioica (L.) K.Koch* |
| *Hamamelis virginiana* |  | *Hamamelis virginiana L.* |
| *Ilex aquifolium* |  | *Ilex aquifolium L.* |
| *Ilex opaca* | unresolved name | *Ilex (opaca)* |
| *Juglans cinerea* |  | *Juglans cinerea L.* |
| *Juglans microcarpa* |  | *Juglans microcarpa Berland.* |
| *Juglans nigra* |  | *Juglans nigra L.* |
| *Juglans regia* |  | *Juglans regia L.* |
| *Juniperus ashei* |  | *Juniperus ashei J.Buchholz* |
| *Juniperus communis* |  | *Juniperus communis L.* |
| *Juniperus oxycedrus* |  | *Juniperus oxycedrus L.* |
| *Juniperus silicicola* | synonym | *Juniperus virginiana var. silicicola (Small) A.E.Murray* |
| *Juniperus virginiana* |  | *Juniperus virginiana L.* |
| *Larix decidua* |  | *Larix decidua Mill.* |
| *Larix laricina* |  | *Larix laricina (Du Roi) K.Koch* |
| *Laurus nobilis* |  | *Laurus nobilis L.* |
| *Liquidambar styraciflua* |  | *Liquidambar styraciflua L.* |
| *Liriodendron tulipifera* |  | *Liriodendron tulipifera L.* |
| *Maclura pomifera* |  | *Maclura pomifera (Raf.) C.K.Schneid.* |
| *Magnolia acuminata* |  | *Magnolia acuminata (L.) L.* |
| *Magnolia fraseri* |  | *Magnolia fraseri Walter* |
| *Magnolia grandiflora* |  | *Magnolia grandiflora L.* |
| *Magnolia macrophylla* |  | *Magnolia macrophylla Michx.* |
| *Magnolia virginiana* |  | *Magnolia virginiana L.* |
| *Malus sylvestris* |  | *Malus sylvestris Mill.* |
| *Morus rubra* |  | *Morus rubra L.* |
| *Nyssa aquatica* |  | *Nyssa aquatica L.* |
| *Nyssa ogeche* |  | *Nyssa ogeche Bartram ex Marshall* |
| *Nyssa sylvatica* |  | *Nyssa sylvatica Marshall* |
| *Olea europaea* |  | *Olea europaea L.* |
| *Ostrya carpinifolia* |  | *Ostrya carpinifolia Scop.* |
| *Ostrya virginiana* |  | *Ostrya virginiana (Mill.) K.Koch* |
| *Oxydendrum arboreum* |  | *Oxydendrum arboreum (L.) DC.* |
| *Persea borbonia* |  | *Persea borbonia (L.) Spreng.* |
| *Phillyrea latifolia* |  | *Phillyrea latifolia L.* |
| *Picea abies* |  | *Picea abies (L.) H.Karst.* |
| *Picea glauca* |  | *Picea glauca (Moench) Voss* |
| *Picea mariana* |  | *Picea mariana (Mill.) Britton, Sterns & Poggenb.* |
| *Picea rubens* |  | *Picea rubens Sarg.* |
| *Pinus banksiana* |  | *Pinus banksiana Lamb.* |
| *Pinus echinata* |  | *Pinus echinata Mill.* |
| *Pinus elliottii* |  | *Pinus elliottii Engelm.* |
| *Pinus glabra* |  | *Pinus glabra Walter* |
| *Pinus mugo* |  | *Pinus mugo Turra* |
| *Pinus nigra* |  | *Pinus nigra J.F.Arnold* |
| *Pinus palustris* |  | *Pinus palustris Mill.* |
| *Pinus pinaster* |  | *Pinus pinaster Aiton* |
| *Pinus pinea* |  | *Pinus pinea L.* |
| *Pinus pungens* |  | *Pinus pungens Lamb.* |
| *Pinus resinosa* |  | *Pinus resinosa Aiton* |
| *Pinus rigida* |  | *Pinus rigida Mill.* |
| *Pinus serotina* |  | *Pinus serotina Michx.* |
| *Pinus strobiformis* |  | *Pinus strobiformis Engelm.* |
| *Pinus strobus* |  | *Pinus strobus L.* |
| *Pinus sylvestris* |  | *Pinus sylvestris L.* |
| *Pinus taeda* |  | *Pinus taeda L.* |
| *Pinus virginiana* |  | *Pinus virginiana Mill.* |
| *Pistacia lentiscus* |  | *Pistacia lentiscus L.* |
| *Planera aquatica* |  | *Planera aquatica J.F.Gmel.* |
| *Platanus occidentalis* |  | *Platanus occidentalis L.* |
| *Populus alba* |  | *Populus alba L.* |
| *Populus balsamifera* |  | *Populus balsamifera L.* |
| *Populus deltoides* |  | *Populus deltoides W. Bartram ex Marshall* |
| *Populus grandidentata* | synonym | *Populus tremula subsp. grandidentata (Michx.) Á. Löve & D. Löve* |
| *Populus heterophylla* | unresolved name | *Populus (heterophylla)* |
| *Populus nigra* |  | *Populus nigra L.* |
| *Populus tremula* |  | *Populus tremula L.* |
| *Populus tremuloides* |  | *Populus tremuloides Michx.* |
| *Prunus americana* | synonymy not resolvable without specific variety | *Prunus (americana)* |
| *Prunus avium* |  | *Prunus avium L.* |
| *Prunus mahaleb* |  | *Prunus mahaleb L.* |
| *Prunus nigra* | synonym | *Armeniaca dasycarpa (Ehrh.) Borkh.* |
| *Prunus padus* |  | *Prunus padus L.* |
| *Prunus pensylvanica* | unresolved name | *Prunus (pensylvanica)* |
| *Prunus serotina* |  | *Prunus serotina Ehrh.* |
| *Prunus virginiana* | synonym | *Padus virginiana (L.) M. Roem.* |
| *Ptelea trifoliata* |  | *Ptelea trifoliata L.* |
| *Pyrus communis* |  | *Pyrus communis L.* |
| *Quercus alba* |  | *Quercus alba L.* |
| *Quercus bicolor* |  | *Quercus bicolor Willd.* |
| *Quercus cerris* |  | *Quercus cerris L.* |
| *Quercus coccinea* |  | *Quercus coccinea Münchh.* |
| *Quercus durandii* | synonym | *Quercus sinuata Walter var. sinuata* |
| *Quercus ellipsoidalis* |  | *Quercus ellipsoidalis E.J.Hill* |
| *Quercus ilex* |  | *Quercus ilex L.* |
| *Quercus ilicifolia* |  | *Quercus ilicifolia Wangenh.* |
| *Quercus imbricaria* |  | *Quercus imbricaria Michx.* |
| *Quercus incana* |  | *Quercus incana Bartram* |
| *Quercus laevis* |  | *Quercus laevis Walter* |
| *Quercus laurifolia* |  | *Quercus laurifolia Michx.* |
| *Quercus lyrata* |  | *Quercus lyrata Walter* |
| *Quercus macrocarpa* |  | *Quercus macrocarpa Michx.* |
| *Quercus marilandica* |  | *Quercus marilandica (L.) Münchh.* |
| *Quercus michauxii* |  | *Quercus michauxii Nutt.* |
| *Quercus muehlenbergii* |  | *Quercus muehlenbergii Engelm.* |
| *Quercus nigra* |  | *Quercus nigra L.* |
| *Quercus nuttallii* | synonym | *Quercus texana Buckley* |
| *Quercus oglethorpensis* |  | *Quercus oglethorpensis W.H.Duncan* |
| *Quercus palustris* |  | *Quercus palustris Münchh.* |
| *Quercus petraea* |  | *Quercus petraea (Matt.) Liebl.* |
| *Quercus phellos* |  | Quercus phellos L. |
| *Quercus prinus* | synonym | Quercus michauxii Nutt. |
| *Quercus pubescens* |  | Quercus pubescens Willd. (Q. Humilis) |
| *Quercus robur* |  | Quercus robur L. |
| *Quercus rubra* |  | Quercus rubra L. |
| *Quercus shumardii* |  | Quercus shumardii Buckley |
| *Quercus stellata* |  | Quercus stellata Wangenh. |
| *Quercus suber* |  | Quercus suber L. |
| *Quercus velutina* |  | Quercus velutina Lam. |
| *Quercus virginiana* |  | Quercus virginiana Mill. |
| *Robinia pseudoacacia* |  | Robinia pseudoacacia L. |
| *Salix alba* |  | Salix alba L. |
| *Salix amygdaloides* |  | Salix amygdaloides Andersson |
| *Salix caprea* |  | Salix caprea L. |
| *Salix fragilis* |  | Salix fragilis L. |
| *Salix nigra* |  | Salix nigra Marshall |
| *Salix pentandra* |  | Salix pentandra L. |
| *Salix purpurea* |  | Salix purpurea L. |
| *Sambucus nigra* |  | Sambucus nigra L. |
| *Sapindus drummondii* |  | Sapindus drummondii Hook. & Arn. |
| *Sassafras albidum* |  | Sassafras albidum (Nutt.) Nees |
| *Sorbus americana* | synonym | Aucuparia americana (Marshall) Nieuwl. |
| *Sorbus aria* |  | Sorbus aria (L.) Crantz |
| *Sorbus aucuparia* |  | Sorbus aucuparia L. |
| *Sorbus domestica* |  | Sorbus domestica L. |
| *Sorbus torminalis* |  | Sorbus torminalis (L.) Crantz |
| *Taxodium distichum* |  | Taxodium distichum (L.) Rich. |
| *Taxus baccata* |  | Taxus baccata L. |
| *Thuja occidentalis* |  | Thuja occidentalis L. |
| *Tilia americana* |  | Tilia americana L. |
| *Tilia cordata* |  | Tilia cordata Mill. |
| *Tilia heterophylla* | synonym | Tilia americana var. heterophylla (Vent.) Loudon |
| *Tilia platyphyllos* |  | Tilia platyphyllos Scop. |
| *Tsuga canadensis* |  | Tsuga canadensis (L.) Carrière |
| *Tsuga caroliniana* |  | Tsuga caroliniana Engelm. |
| *Ulmus alata* |  | Ulmus alata Michx. |
| *Ulmus americana* |  | Ulmus americana L. |
| *Ulmus crassifolia* |  | Ulmus crassifolia Nutt. |
| *Ulmus glabra* |  | Ulmus glabra Huds. |
| *Ulmus laevis* |  | Ulmus laevis Pall. |
| *Ulmus minor* |  | Ulmus minor Mill. |
| *Ulmus rubra* |  | Ulmus rubra Muhl. |
| *Ulmus serotina* |  | Ulmus serotina Sarg. |
| *Ulmus thomasii* |  | Ulmus thomasii Sarg. |
