# Supplementary material for: Functional Resilience against Climate-Driven Extinctions – Comparing the Functional Diversity of European and North American Tree Floras
Source: PLoS One. 2016 Feb 5;11(2):e0148607. doi: 10.1371/journal.pone.0148607 (PMC4743854; doi:10.1371/journal.pone.0148607)
Supplement: S3 Table — (DOCX) [file pone.0148607.s011.docx]

# Appendix S3 Table - Trait description

|  | trait name | trait description |
| --- | --- | --- |
| leaf traits | |  |
|  | leaf area | the area of the leaf surface for single leaved leaves and of all  leaflets on a rachis for compound leaves |
|  | leaf arrangement | describes how leaves are arranged at their nodes |
|  | leaf carbon:nitrogen ratio | the percentage of organic carbon divided by the percentage of total  nitrogen in organic material; organic material is specified as the  above ground herbaceous material of a woody plant |
|  | leaf composition | describes whether a leaf is compound consisting of several leaflets  on a rachis or whether it is single-leaved with one leaf on a etiole |
|  | leaf margin | the shape of the leaf margin |
|  | leaf type | the shape of a leaf in combination whether it is deciduous or  evergreen |
|  | specific leaf area | leaf area per leaf weight |
|  |  |  |
| plant level traits | |  |
|  | fire resistance (flammability) | to the ability of a species to resist burning or alternatively to carry a  fire; fire resistant means low flammable |
|  | growth form | defines a tree as a perennial upright woody plant able to reach at  least 6 m in height, a shrub as a woody plant with multiple stems  and lower height, usually less than 5–6 m (15–20 ft) tall and  intermediate between tree and shrub when both growth forms  are combined or even likely |
|  | growth rate | the capability of a species to produce aboveground biomass  compared to other tree species |
|  | lifespan | the expected lifespan of a tree species relative to other tree species |
|  | maximum height | maximum height observed at a given site |
|  | nitrogen fixation | the amount of nitrogen which is fixed by a species in monoculture |
|  | potential allelopathy | the chemical potential of a species to be considered allelopathic;  the relative ranking of species are based upon the completeness  of the allelopathic literature, of species’ growth strategies,  successional position, and conjecture of the author |
|  | resprout ability after disturbance | the ability of a species to resprout after aboveground biomass  removal |
|  | resprout ability after fire | the ability of a species to resprout after fire |
|  | toxicity | the relative toxicity of the plant to either humans or livestock |

|  | trait name | trait description |
| --- | --- | --- |
| root and wood traits | |  |
|  | are tracheids present? | are tracheids present in hardwood? |
|  | bark surface | roughness of the bark |
|  | conduit type and arrangement (porosity) | the arrangement of the vessels (exclusively angiosperms) throughout a growing season or whether there are tracheids  (exclusively gymnosperms) instead |
|  | rooting habit | root habit with respect to rooting depth |
|  | wood density | oven dry mass per fresh volume |
| reproduction traits | |  |
|  | dispersal syndrome | the way how the plant disperses its seeds |
|  | seed mass | seed dry weight |
|  | seed spread rate | the capability of a species to spread through its seed production  compared to other tree species |
|  | vegetative spread rate | the capability of a species to spread compared to other tree species |
